# The reliability of molecular dynamics simulations of the multidrug transporter P-glycoprotein in a membrane environment

**DOI:** 10.1371/journal.pone.0191882

**Published:** 2018-01-25

**Authors:** Karmen Condic-Jurkic, Nandhitha Subramanian, Alan E. Mark, Megan L. O’Mara

**Affiliations:** 1 Research School of Chemistry, The Australian National University, Canberra, ACT 2601, Australia; 2 School of Chemistry and Molecular Biosciences, University of Queensland, Brisbane, ACT 4072, Australia; Universidade Nova de Lisboa Instituto de Tecnologia Quimica e Biologica, PORTUGAL

## Abstract

Despite decades of research, the mechanism of action of the ABC multidrug transporter P-glycoprotein (P-gp) remains elusive. Due to experimental limitations, many researchers have turned to molecular dynamics simulation studies in order to investigate different aspects of P-gp function. However, such studies are challenging and caution is required when interpreting the results. P-gp is highly flexible and the time scale on which it can be simulated is limited. There is also uncertainty regarding the accuracy of the various crystal structures available, let alone the structure of the protein in a physiologically relevant environment. In this study, three alternative structural models of mouse P-gp (3G5U, 4KSB, 4M1M), all resolved to 3.8 Å, were used to initiate sets of simulations of P-gp in a membrane environment in order to determine: a) the sensitivity of the results to differences in the starting configuration; and b) the extent to which converged results could be expected on the times scales commonly simulated for this system. The simulations suggest that the arrangement of the nucleotide binding domains (NBDs) observed in the crystal structures is not stable in a membrane environment. In all simulations, the NBDs rapidly associated (within 10 ns) and changes within the transmembrane helices were observed. The secondary structure within the transmembrane domain was best preserved in the 4M1M model under the simulation conditions used. However, the extent to which replicate simulations diverged on a 100 to 200 ns timescale meant that it was not possible to draw definitive conclusions as to which structure overall was most stable, or to obtain converged and reliable results for any of the properties examined. The work brings into question the reliability of conclusions made in regard to the nature of specific interactions inferred from previous simulation studies on this system involving similar sampling times. It also highlights the need to demonstrate the statistical significance of any results obtained in simulations of large flexible proteins, especially where the initial structure is uncertain.

## Introduction

P-glycoprotein is a member of a large family of membrane-bound ATP-Binding Cassette (ABC) transporters, which utilise ATP hydrolysis to induce a conformational change to transport a variety of substrates across the membrane [1–3]. P-glycoprotein (P-gp) has been implicated in multidrug resistance of cancer cells, and is able to efflux a diverse range of neutral and cationic xenobiotics, varying in size and chemical properties [4–6]. Despite being one of the most comprehensively studied ABC transporters, many aspects of the P-gp transport mechanism remain elusive, including how P-gp binds such a wide range of substrates, and how ATP hydrolysis drives the conformational change that ultimately expels substrate from the cell [7, 8].

**Fig 1:**
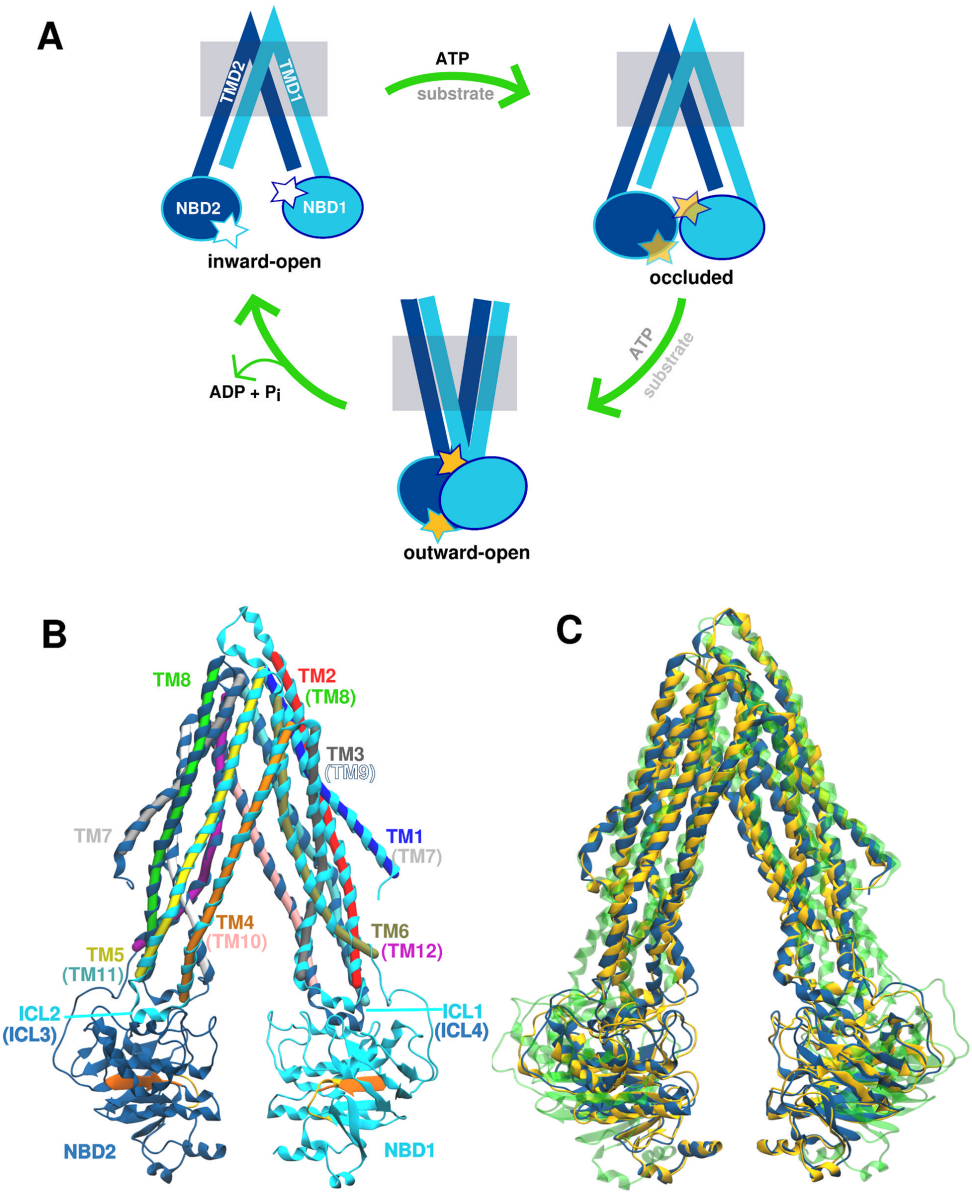
Putative transport cycle of P-glycoprotein. **(A)** The transport cycle is assumed to start from the inward-open conformation, followed by the occluded state assumed to form upon ATP and/or substrate binding. Finally, the transporter adopts the outward-open conformation driven by ATP hydrolysis and subsequently, the substrate is released in the extracellular space. **(B)** The inward-open conformation of crystallised mouse P-gp structure (4M1M) contains 12 transmembrane helices (TM1-12) and two nucleotide binding domains (NBDs). Helices TM1-6 and NBD1 are shown in light blue, while helices TM7-12 and NBD2 are given in dark blue shade. The conserved residues involved in ATP binding and hydrolysis, Walker A and signature motifs, are highlighted in orange and yellow, respectively (see S1 Fig for the back view). **(C)** Overlap of the three crystal structure models of mouse P-gp solved at 3.8 Å: 3G5U (yellow), 4KSB (green) and 4M1M (blue). Differences in the secondary structure assignment of TM helices between 3G5U and 4M1M are visible, as well as wider separation between NBDs found in 4KSB model.

The initial elucidation of homologous bacterial ABC transporter crystal structures [9–11], solved in different conformations, has made a major contribution to our understanding of the P-gp transport mechanism. These structures have provided a basis for construction of a putative transport cycle (Fig 1A). In this cycle, substrate binds to the transmembrane pore when the transporter is in an inward-facing (IF) conformation, in which the transmembrane pore is occluded to the extracellular environment and open to the cytoplasm. The conformational changes associated with ATP binding and hydrolysis lead to an outward-facing (OF) state in which the pore is open to the extracellular space, while the cytoplasmic entrance is occluded.

In addition to bacterial transporters, a number of crystal structures of homologous eukaryotic ABC transporters have been solved [12–14], including several different structures of P-glycoprotein from mouse [15–18] and *C. elegans* [19]. The structure of human P-gp has not been solved thus far, but it shares 87% sequence identity with its murine counterpart. An example of an inward-open crystallographic conformation of mouse P-gp is shown in Fig 1B (PDB ID: 4M1M [16]), which shares a common architecture with the rest of the B-subfamily of ABC transporters and has two (pseudo)symmetrical halves with domain swapping features (Fig 1B). Each half consists of one transmembrane domain (TMD) containing six transmembrane (TM) helices and one nucleotide binding domain (NBD). Each NBD contains the highly conserved Walker A and signature motif sequences that interact to form two catalytic ATP binding sites upon head-to-tail dimerization of the NBDs. This interaction has been proposed to result in a conformational change to the outward-open conformation [20]. With the exception of two bacterial transporters, Sav1866 [10] and MsbA [11], which crystallised in an outward-open conformation, all available structures of ABC exporters are in an inward-open conformation. The conformation of P-gp under physiological conditions is still an open debate as membrane protein purification usually involves extraction from the native membrane into detergent micelles or artificial lipid bilayers, which may result in improper folding, denaturation, or loss of activity [21]. The choice of detergent can influence both the protein conformation [22, 23] as well as crystallisation outcomes [24], such as the internal order of the crystal. Furthermore, crystal packing may select for conformations that readily form a crystal lattice, putting a strain on the structure and challenging the assumption that the crystal structure is always closely related to a biologically active conformation [25, 26].

Molecular dynamics (MD) simulations are increasingly used to explore protein structure and dynamics at atomic resolution [27, 28]. In such simulations, proteins can sample different conformations under given conditions, and provide insights into structure-function relationships. A large number of MD studies have been performed on mouse P-gp, as well as on homology models of human P-gp based either on mouse P-gp or homologous ABC transporters. The various aims of these studies were to gain a better understanding of the protein dynamics in the membrane environment [29–34], examine the effect of ATP and substrate binding [35–38], or to identify key residues involved in substrate binding [39, 40]. None of the studies reported any spontaneous conformational change from the inward- to outward-facing conformation, although several targeted MD simulations were used to investigate potential transition pathways between these two conformations [41, 42]. Despite such studies, the molecular mechanism of P-gp remains poorly understood. The difficulty is that both simulations and indeed different experiments yield apparently contradictory results. In fact, one of the few findings that have emerged from both the simulations and experimental data is that the structure of P-gp is highly flexible. To date, MD simulations, EPR and NMR studies of P-gp all suggest that it is best represented by a dynamic ensemble of structures, rather than a series of distinct conformational snapshots [43–49]. The conformational dynamics within P-gp are expected due to the large-scale conformational changes required for the proposed transport cycle, although findings such as the unfolding of certain helices were reported in several MD studies. The observed unfolding has been attributed either to the relatively high Gly/Pro content of the TMDs [46], or the instability of the crystal structure under the simulated conditions [32]. However, these findings also might have been simply simulation artefacts.

There are more than 20 crystal structures of mouse P-gp currently deposited in Protein Data Bank [15–18, 50]. These have been solved under varying crystallisation conditions, with dataset resolutions ranging from 3.4–4.4 Å. While these lower-resolution datasets (3–4 Å) can provide information about secondary structure motifs, the positions of the side-chains and any ligands are difficult to assign precisely. This leads to increased uncertainty regarding the atomic positions [51], and potentially to local structural instabilities during MD simulations [52, 53]. This is especially relevant in the context of the original 2009 mouse P-gp structure (PDB ID: 3G5U), solved to 3.8 Å resolution [15], which was subjected to revision after the publication of a *C. elegans* P-gp crystal structure (resolved to 3.4 Å) revealed discrepancies between the two models [19]. These discoveries led to the re-refinement of the original diffraction dataset [16], resulting in an alternate structural model of P-gp (PDB ID: 4M1M) with better structure refinement parameters, summarised in Table 1. The most notable difference between the original 3G5U model and the revised 4M1M model is the change in the registry of four of the twelve transmembrane helices (TM), namely TM3, TM4, TM5, and TM12 (Fig 1B and 1C). A comprehensive description of the differences between 3G5U and 4M1M P-gp can be found in Li et al [16].

**Table 1:** Refinement statistics for the three P-glycoprotein crystallographic models [15–17].

Crystallographic refinement statistic	3G5U	4M1M	4KSB
Resolution range high (Å)	3.8	3.8	3.8
Resolution range low (Å)	19.98	24.89	92.56
Number of reflections	41131	41385	21753
R value (working set)	0.306	0.267	0.357
Free R value	0.346	0.267	0.357
Free R value test set	4203	4203	1062
Estimated coordinate error (maximum likelihood)	-	0.46	0.82
Phase error (degrees, maximum-likelihood)	-	27.47	38.35
Luzzati plot	0.86	-	-
SigmaA	0.82	-	-
Ramachandran outliers (asymmetric unit)	594	92	79
Atomic coordinates in asymmetric unit	18352	18378	9171

A third structural model of the *apo* P-gp (PDB ID: 4KSB), also resolved to 3.8 Å, was published around the same time as the 4M1M model, but derived from an independent set of crystallographic data [17]. The 4KSB model was crystallised using a different protocol to that of 3G5U/4M1M and was subjected to reductive methylation to promote crystallisation. The structure refinement parameters for the 4KSB model were close to that of the 4M1M model, however a wider separation between the nucleotide binding domains (NBDs) was proposed in the 4KSB structural model (Fig 1C). Changes in the helical registry were again noted in TM3, TM4, TM5, and TM12, when compared to the original 3G5U structure. The 4KSB model also includes a registry shift in the same four TM helices, however it is notable that the amino acid assignment in TM12 differs to that of 4M1M model. This results in three unique structural models of *apo* P-gp at 3.8 Å resolution (Fig 1C). Ideally, MD simulations initiated from any one of these crystallographic models should sample a similar region of conformational space, provided the MD simulations were conducted under the same conditions and of sufficient duration. However, such conformational changes may not be sampled on currently accessible time scales, which range from nanoseconds to microseconds [54].

The majority of the simulations published to date have been based on trajectories in the order of 100 ns, using the 3G5U crystal structure as the starting conformation. The question is, given the inherent flexibility of P-gp and the uncertainties in the structure of P-gp, can the results of such simulations be considered reliable? Here we examine the conformational space sampled by simulations initiated from the three P-gp structures corresponding to PDB entries 3G5U, 4M1M and 4KSB, each of which contain differences in their residue assignment. We compare the results of 9 independent 200 ns MD simulations initiated from the three different 3.8 Å resolution P-gp structures embedded in a membrane environment, following a standard simulation protocols for membrane proteins. The protein dynamics, structural integrity and backbone fluctuations of each systems were examined across all 9 simulations. The aim is to determine which of the features extracted from the simulations, if any, are observed in all three models, and which starting configuration could be considered most reliable under the simulation conditions examined.

## Results

### Protein dynamics

A set of three replicate simulations of 200 ns duration were performed for each of the membrane-embedded, solvated P-gp systems corresponding to the three alternative structural models (PDB ID: 3G5U, 4M1M, and 4KSB). The simulations did not include the linker (∼ 60 residues), which was unresolved in all three crystal structures. The root mean squared deviations (RMSD) of the backbone atoms from the positions in the starting crystal structure were calculated for each simulation (S2 Fig). In each of the nine simulations, the backbone RMSDs showed a rapid increase in the first 10 ns, followed by more gradual rise over the course of the simulation. The final backbone RMSDs ranged from 0.7 nm to 1.0 nm across the nine simulations. Visual inspection showed that these large deviations were the result of systematic conformational changes. Most notably, in all simulations, the separation between the NBDs decreased over time in comparison to the crystal structure. The average backbone RMSD of the individual NBDs ranged between 0.3–0.4 nm (S3 Fig), mostly due to variations in the disordered loops. There were no significant differences in the structural stability of the NBDs of each of the P-gp structural models. The average backbone RMSD of the TMD was higher than that of the isolated globular NBDs, ranging between 0.4 and 0.5 nm with respect to the initial starting structure over the nine simulations. The disparity between the RMSDs measured for the entire system, and each domain is consistent with the conformational rearrangement of the domains observed visually (S3 Fig). In all three P-gp systems, this conformational change involves the pivoting of the NBDs inward to form a protein-protein interface and a narrowing of the Λ-shaped conformation of the TMD in the 4KSB systems.

**Fig 2:**
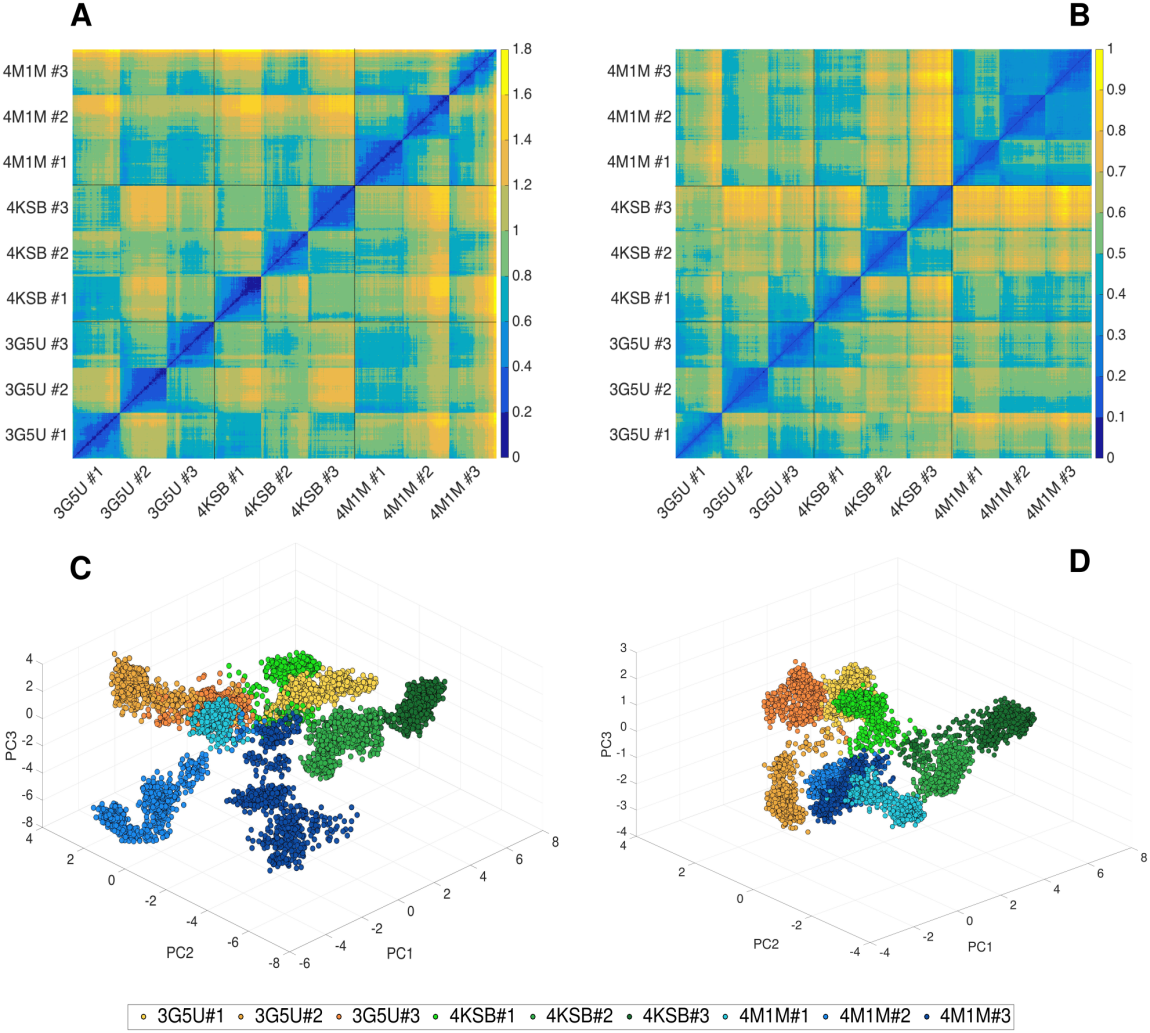
Pairwise RMSD and principal component analysis (PCA). Heatmap representing pairwise RMSD [nm] calculated for the backbone atoms of P-gp conformations sampled in all 9 trajectories started from 3G5U, 4KSB and 4M1M for **(A)** the entire protein and **(B)** the TMD coordinate subset only. Black gridlines separate the three systems, while each replica (3G5U#1-3, 4KSB#1-3, 4M1M#1-3) is visible as a square formation around diagonal line (diagonal corresponds to the RMSD of a structure to itself, which is 0 nm). Conformational space sampled in each simulation as a function of the first three principal components (PC1, PC2, PC3) obtained from the analysis of the concatenated 9 trajectories for (**C**) the entire protein and (**D**) the TMD only, respectively. Each replica is shown in a different shade of yellow (3G5U), green (4KSB) or blue (4M1M). The TMD residues used in the pairwise RMSD and PC analysis were Tyr41-Ala358 (TMD1) and Leu684-Phe990 (TMD2).

**Fig 3:**
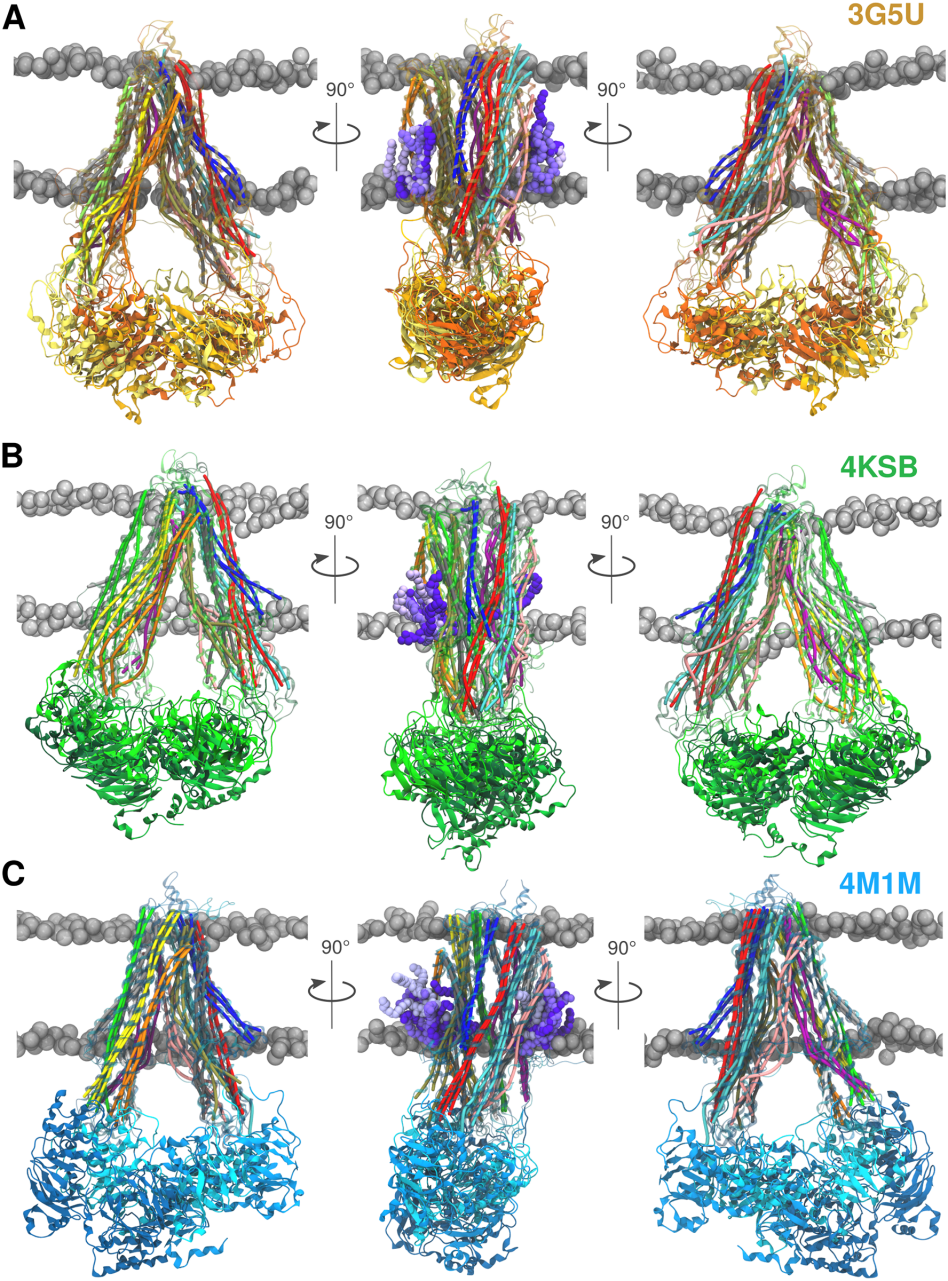
Final snapshots. Overlap of the final P-gp conformations after 200 ns obtained from three replicas performed for each system. Each replica is coloured in a different shade of yellow for 3G5U **(A)**, green for 4KSB **(B)**, and blue for the 4M1M **(C)** system, where the lightest shade corresponds to replica #1 and the darkest to replica #3. The left and right panels show the TMD1 and the TMD2 at the front, respectively. The middle panel gives the side-view with lipids interacting with the TM4/6 (left side) and the TM10/12 (right side) portal. The POPC molecules are shown in different shades of violet and again, the lightest shade corresponds to replica #1. The protein is shown in cartoon representation and the TM helices are represented as cylinders that follow the helix axis (*bendices*) to highlight the change in helical geometry [55]. The POPC/cholesterol bilayer is depicted using the VDW sphere representation of phosphate atoms (grey).

The backbone RMSD values measured for each replica using the respective starting crystal structures as a reference are comparable, suggesting that the extent of structural change is similar in all three systems. To determine whether all systems converge to a similar conformation, the pairwise RMSD was calculated across the 9 simulation trajectories. Fig 2A shows the backbone RMSD between pairs of structures corresponding to each frame of the nine trajectories presented as a 2D heat map. The diagonal dark blue line represents the backbone RMSD of a structure to itself and thus has an RMSD of 0 nm. Pairs of structures with the greatest backbone RMSD are in yellow. The heat map shown in Fig 2A reveals that for replicas of the same system, the pairwise RMSDs of conformations range from 0.4–1.4 nm, indicating a variety of conformations are adopted by P-gp in the different replicas. A similar range of pairwise RMSDs was measured between conformations from three the different systems. Here the minimum pairwise RMSD was 0.6 nm, and the maximum was 1.8 nm. The overlap of the final three conformations obtained for each system is shown in Fig 3, illustrating the degree of conformational diversity. The main conformational differences can be attributed to the movement of the NBDs. Due to the differences in TMDs between the three structural models, the pairwise backbone RMSDs were calculated for the TMD only to examine the similarity of these domains between three systems. Fig 2B shows a heat map corresponding to just the TMDs. The TMD in the 4M1M system shows less variation (lower RMSD) between replicates compared to either 3G5U or 4KSB.

Similar results were obtained from a principle component (PC) analysis performed on the concatenated trajectory containing all 9 simulations generated from the three crystal structures. The major contribution to the first principal component (PC1), which is the eigenvector containing the largest variance and accounts for 34% of protein motion, originates from the motion of the NBDs (S4 Fig), while the greatest fluctuations of the TMD were found in the intracellular loops ICL1 and ICL4 connecting TM2-TM3 and TM10-TM11, respectively. Principal components corresponding to the second (PC2) and third (PC3) largest variance have a similar intensity in the TMD region, while the NBD contributions are reduced (S4 Fig). The fraction of variance corresponding to PC2 and PC3 is 15% and 10%, respectively, for both the total protein motion and the TMD only. The contributions of all 10 calculated PCs are given in S5 Fig, including the values corresponding to the PCs calculated for each replica separately. Pairwise comparisons between the principal components obtained from each replica and from the concatenated trajectory are provided in S1, S2 and S3 Tables for the 3G5U, 4KSB and 4M1M models, respectively. Plotting this concatenated trajectory as a function of the first three principal components (Fig 2C) reveals that the distribution of P-gp conformations differs not only between the simulations launched from the different crystal structures, but also between replicas of the same system. Trajectories initiated from 3G5U are shown in orange, 4KSB in green and 4M1M in blue tones. The data points corresponding to the same trajectory are broadly clustered together, and there is little overlap between any of the trajectories in this space, also confirmed by the low covariance overlap calculated for each pair of all the replicas (S4 Table). This result suggests that the region of conformational space explored by the protein in each simulation is unique. This supports the view that P-gp is a very flexible and dynamic system. However, it is not possible to distinguish between the intrinsic dynamics and other factors that might influence the observed motion, such as the local quality of the model, the removal of crystal packing forces, change in the environment, etc.

PC analysis was also repeated using just the coordinates of the TMDs to remove the effects of the NBDs, which undergo large-scale movements, to understand the extent and the location of the structural changes of the TMDs in more detail. The results (Fig 2B and 2D) again show marked differences between the simulations initiated using each of the three structural models. There are also marked differences between the three replicas initiated using 3G5U and 4KSB. In contrast, the three replicas initiated using 4M1M yielded very similar results in agreement with the pairwise RMSD calculations. It is important to note that the variance in the TMDs is lower compared to the entire protein, as expected (S4 Fig). Finally, the RMS fluctuations were measured for the C*α* atoms in the entire protein and TMDs only (S6 Fig), showing similar patterns between three systems—the greatest mobility corresponds to NBDs, the intracellular loops (ICL1-4) and the extracellular loop connecting TM1 and TM2. The RMS fluctuations for the entire protein are comparable between the three systems, but the smallest TMDs fluctuations were found in 4M1M system. All this suggests that the 4M1M TMDs are more structurally stable in the simulations than either 3G5U or 4KSB.

### Transmembrane domains

**Fig 4:**
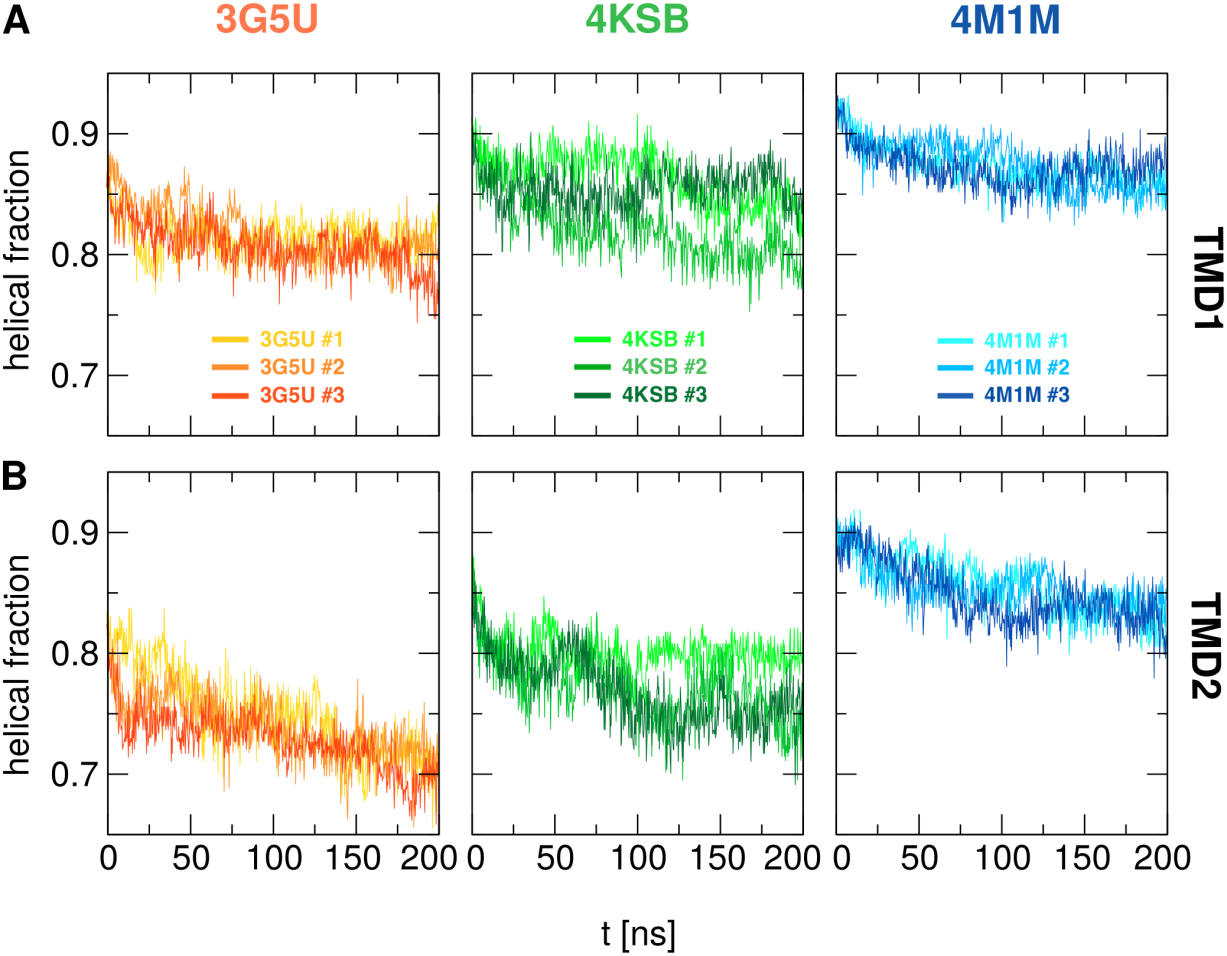
Helical content of transmembrane domain. The fraction of helical content in TMD1 **(A)** and TMD2 **(B)** as function of time for each replica started from 3G5U, 4KSB and 4M1M models calculated from the secondary structure analysis performed using the DSSP algorithm.

The most notable differences between the amino acid assignment in the 3G5U, 4M1M, and 4SKB structural models are in the TM helices 3, 4, 5 and 12. In addition to these differences, 4KSB features a wider angle between the TMD helices (Fig 1C). For each simulation, the backbone RMSD calculated with respect to its starting crystal structure was 0.4–0.5 nm (S2 and S3 Figs), suggesting that the scale of the structural changes within the TMDs is comparable for all three systems during each 200 ns simulation. However, the nature of these conformational changes was different in each simulation. The secondary structure content of TMD1 (TM1-6) and TMD2 (TM7-12) was assessed using the DSSP algorithm and the fraction of helical content was calculated as a function of time, shown in Fig 4. The average values for each run are given in Table 2.

**Table 2:** Calculated average helical content (and standard deviation) from DSSP analysis of TMD1 (TM1-6) and TMD2 (TM7-12) for each replica of simulated systems 3G5U, 4KSB and 4M1M. The amount of helical content was also averaged over all three simulations. TMD1 spans residues Trp44-Asp366, while TMD2 spans residues Trp704-Thr1011.

Helicity	TMD1			TMD2		
	3G5U	4KSB	4M1M	3G5U	4KSB	4M1M
**Crystal structure**	0.87	0.90	0.92	0.83	0.85	0.90
**Replica #1**	0.82 ± 0.02	0.86 ± 0.02	0.87 ± 0.02	0.75 ± 0.04	0.80 ± 0.02	0.86 ± 0.02
**Replica #2**	0.82 ± 0.02	0.82 ± 0.02	0.87 ± 0.02	0.74 ± 0.03	0.77 ± 0.03	0.86 ± 0.02
**Replica #3**	0.81 ± 0.02	0.85 ± 0.02	0.87 ± 0.02	0.73 ± 0.02	0.77 ± 0.03	0.85 ± 0.02
**Total**	0.81 ± 0.02	0.85 ± 0.02	0.87 ± 0.02	0.74 ± 0.03	0.78 ± 0.02	0.85 ± 0.02

The results show that 4M1M has the highest percentage of helical content, both in the starting crystal structure and in the resulting simulations (Table 2). The ranking of the structures based on helical content is 4M1M > 4KSB > 3G5U, while TMD1 has a higher percentage of helical content than TMD2. A certain degree of unfolding was found in all three systems, but it was especially pronounced in the 3G5U system (Table 2. The DSSP plots as function of time reveals loss of helicity in TM4, TM5, TM10, TM11, TM12 and the intracellular coupling loops (ICLs) that form an interface with the NBDs, ICL3 and ICL4 in particular (S7 Fig). The unfolding of the coupling loops was also observed to a lesser extent in the simulations initiated from the 4KSB model, while in the 4M1M simulations the helical motifs in the ICLs were maintained. The degree of unfolding increased progressively over the 200 ns simulation, although the largest drop in helical content occurs in the first 50 ns (Fig 4). The loss of secondary structure resulted in the deformation of the TM helices of both 3G5U and 4KSB and each simulation resulted with a different TMD conformation. The range of TMD conformations is illustrated with the overlay of the final snapshots for the three replicas for 3G5U, 4KSB and 4M1M in Fig 3. The overlay of the three final conformations of the 4M1M (Fig 3C) system shows that the TMDs adopt a consistent conformation with a well-defined shape, while the overlays of the 3G5U and 4KSB conformations show a range of lateral helical displacements.

Despite the relative stability of the 4M1M TMDs, localised regions like the ICLs exhibit a high degree of mobility, as measured by the root mean square fluctuations (RMSF) of C*α* atoms (S6 Fig) and shown on the secondary structure plots (S7 Fig). Increased mobility was observed in the 4M1M simulations within the portal helices (TM4/6, TM10/12). These helices connect the lipid bilayer to the TM cavity and are implicated as potential entry points for substrate uptake. The protrusion of the tails of different lipids into both the TM4/6 and TM10/12 portals was repeatedly observed in the 4M1M simulations. However, the lipid tails protruded deeper into the protein cavity through the TM4/6 portal (Fig 3). Similar lipid tail protrusions through the TM4/6 portal were reported in a previous study using the 3G5U structure [46]. In the simulations initiated using 3G5U and 4KSB in this work, lipids were observed to partly protrude into the TM10/12 portal, but no lipid protruded into the TM4/6 portal. This may be a consequence of the placement of TM6 directly between TM3 and TM4 in the 3G5U and 4KSB structural models. This effectively occludes the entrance to the TM cavity in the starting conformation. The occlusion of the TM4/6 portal was also noted in the study by Ferreira *et al.*, in which the 3G5U model was used to examine the energetics of drug entry through the TM10/12 portal [40]. On the contrary, a similar study of potential drug entry pathways based on the 4M1M structure identified TM4/6 as the most energetically favourable uptake pathway [56]. Differences in the protein-lipid interactions observed in the 3G5U and 4KSB systems may be attributed to the loss of secondary structure in the TM4/6 and TM10/12 portal helices during the simulations, which directly affects the protein-lipid interface.

### Nucleotide binding domains

**Fig 5:**
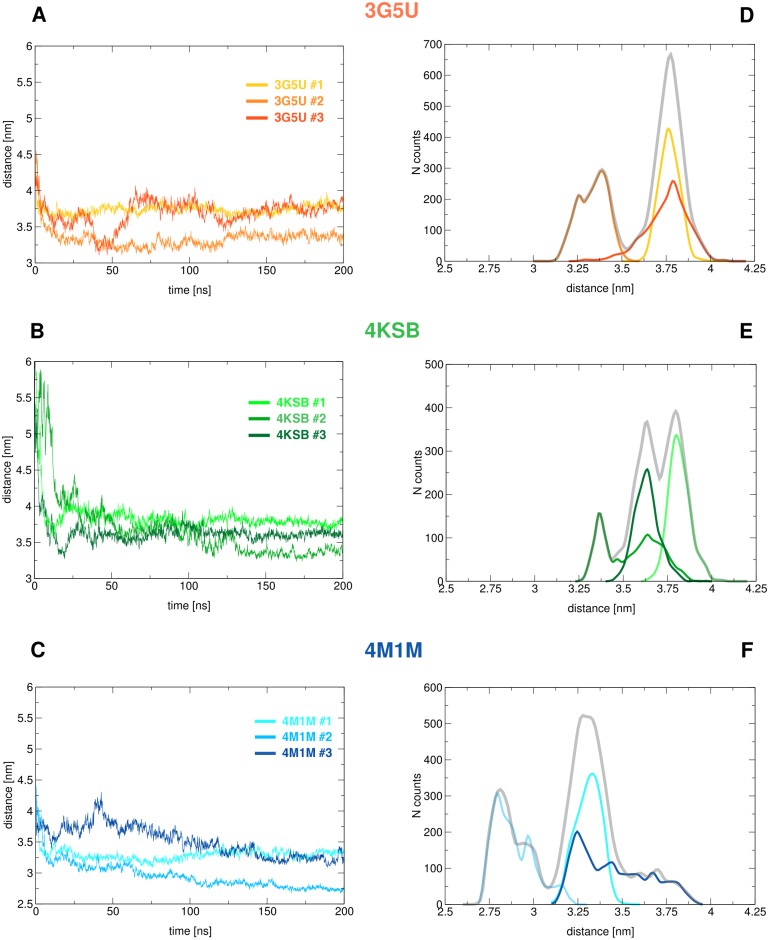
Distances between the NBDs. **(A-C)** Distances measured between the centres of mass (COM) of the nucleotide binding domains during each simulation starting from 3G5U (A), 4KSB (B), and 4M1M (C); **(D-F)** Distribution of the inter-NBD distances obtained for each replica independently and for all three replicas (grey), starting from 3G5U (D), 4KSB (E), and 4M1M (F).

In the simulations performed in this study, all of which were unbiased, there was a rapid association of the nucleotide binding domains. This occurred within the first 10 ns in all cases (Fig 5A–5C). The most pronounced structural change was observed in the 4KSB system, which has the largest inter-NBD separation of the three structural models. After the NBDs had associated, they remained in close contact throughout the simulation. This is consistent with previous MD studies based on the 3G5U structural model [29, 30, 32]. We note, however, that Wen et al. proposed that larger distances are also sampled, based on a combination of MD and DEER experiments [46]. The nature of the NBD1:NBD2 interface in the different simulations is determined by the relative orientation of the NBDs at the time of contact, and the dimer interface is slightly different in each replica of each system. This is illustrated by the inter-NBD distance distribution in Fig 5D–5F. Analysis of the distances between the Walker A motif and Signature motif of opposite NBDs, which form the two ATP binding sites, shows that the association of the NBDs is asymmetric in all three structural models (S8 Fig). This is consistent with previous reports of a non-specific, asymmetric association of the NBDs in the presence and absence of ATP [29, 30, 38, 57]. Fig 3 shows the range of NBD1:NBD2 conformations obtained for each replica initiated using each of the three structural models, after 200 ns of simulation. This conformational diversity suggests that the spread of distances between labelled residues, as observed in DEER experiments, could also arise from conformations in which the NBDs remain in close contact, and do not necessarily imply there is a change in the inter-NBD separation. Other experimental studies involving fluorescence resonance energy transfer (FRET) efficiency measurements [44], intra-NBD cross-linking [58], and electron microscopy projections of membrane-embedded P-gp [59] provide evidence for the NBDs coming into very close contact during the transport cycle. For example, in the case of a homodimeric ABC transporter TM287/288 from *T. maritima* crystallised in the presence and absence of AMP-PNP, the NBDs in both crystal structures were found to be in contact, which was in agreement with supplementary results obtained by DEER measurements [12, 13]. The native conformation of NBDs under physiological conditions perhaps remains inconclusive, but the very dynamic nature of these domains is indisputable.

**Fig 6:**
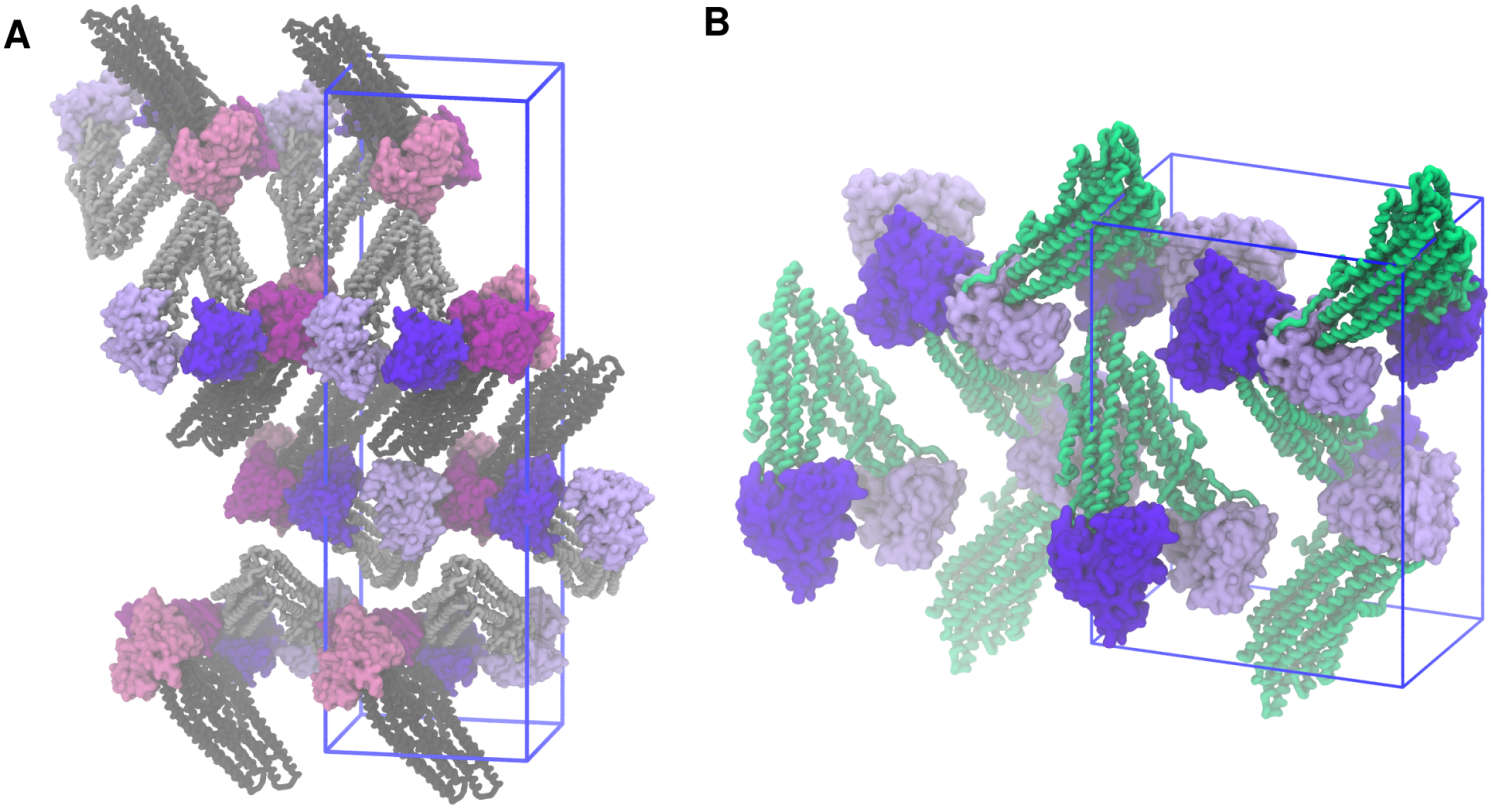
Crystal packing. **(A)** Orientation of P-gp molecules in the crystal lattice from which the 3G5U and 4M1M structural models were derived. The asymmetric unit contains two P-gp molecules (the NBDs highlighted in violet and magenta, respectively). **(B)** The crystal lattice representation of the 4KSB model, where the asymmetric unit contains a single P-gp molecule. Both crystal lattices belong to the P21 21 21 space group and the unit cell is represented with the blue wireframe.

That the formation of a NBD1:NBD2 contact interface was strongly favoured, regardless of whether the starting structure was 3G5U, 4M1M or 4KSB, suggests that the crystallographic conformation could be the result of a combination of the crystallisation conditions and crystal packing forces. Previous simulations have shown that the aggregation of detergent between the NBDs stabilises the splayed 3G5U conformation [29]. Recent crystallographic studies of the P-gp homologue, PglK, provide strong evidence that the crystallization detergent and lattice interactions influence the inter-NBD distance observed in the corresponding protein crystal structures [60]. An examination of the crystal contacts in 3G5U and 4M1M P-gp shows that the NBDs of each P-gp protein are nested in a cavity created by the three surrounding P-gp molecules (Fig 6). In the 4KSB crystal lattice, each NBD contacts the NBD of the neighbouring molecule, while the apex of the TMD of a third protein sits between the NBDs. This suggests that the crystal lattice itself may stabilise the relatively large NBD separations observed in the P-gp crystal structures.

Finally, a hinging motion of the NBDs at the NBD:TMD interface is observed in the simulations initiated using 4M1M, and to a lesser extent, in those initiated using 4KSB (S8 Fig). It is unclear whether this hinging motion of the NBDs is an artefact of the simulation or a true representation of the motion in this region [61]. The 3G5U NBD:TMD interface appears to be more resilient of the three structural models examined. As noted above, there is a loss of helical structure in the ICLs initiated using 3G5U. This also occurred to a lesser extent in the 4KSB system. We assume that the increased disorder in the ICLs allows for the re-adjustment of the TMD:NBD interface in 3G5U, stabilising the interaction.

## Discussion

In this study, three crystal structures of mouse P-gp (PDBid: 3G5U, 4M1M, 4KSB), solved at the same resolution (3.8 Å) were simulated under identical unbiased conditions. In all simulations, the NBDs rapidly made direct contact with each other, favouring direct contact between the NBDs over the large separation found in the crystal structures. This is in line with a number of other simulation studies initiated using both 3G5U ([29, 30, 32, 39]) and 4KSB P-gp [33, 34, 62]. This said, it is also possible that ∼ 60 residue linker region that spans the ∼ 50 Å gap between the C-terminus of NBD1 and the N-terminus of TM7 plays an important role is determining the relative positions of the NBDs and the final NBD1:NBD2 interface. It has been suggested that the linker region may affect the extent of motion of the NBDs [31, 32], but as the structure of this region has not been resolved in any P-gp crystal structure model, regardless of resolution, this conclusion is speculative. As there is little information regarding either the conformation of the linker region or how it lies relative to the rest of the protein, it was not included in these simulations. The scope of this study was to compare the performance of three different starting points without introducing further bias. Note that P-gp remains functional in experiments where the linker has been removed [63].

In this work we have attempted to determine if 200 ns is sufficient to obtained converged structural properties and to determine which of the three selected P-gp structural models, if any, is an appropriate starting point for future simulation studies. Pairwise RMSDs and PC analysis were used to illustrate the extent to which trajectories diverged both as a result of using different starting structures, and also between replica simulations starting from the same structure. However, some common features were observed in all simulations. In particular, the NBDs moved into close contact and adopted an occluded conformation in preference to the open conformation observed in the crystal. The TMD-NBD interface was found to be unstable in the simulations initiated from both 4M1M and 4KSB simulations. This resulted in a hinge motion of the NBDs, but the origin of this behaviour is not clear. These changes may result from the loss of crystal lattice contacts, but they could also reflect shortcomings of the crystallographic model and simulations conditions, such as the missing linker, the choice of the force field and simulation parameters.

The three models had discrepancies between the residue assignments within the TMDs and the simulations showed difference in the degree of secondary structure stability in the TMD between the models. Progressive deformation of the helices (unfolding, kinking, and bending) occurred during the simulations. The TMD helices were most stable in the 4M1M simulations, which showed the highest amount of helical content, while the loss of helicity was most notable in the 3G5U model. The unfolding of the transmembrane helices has been reported in multiple previous studies based on the 3G5U structure. These studies have involved different simulation protocols and a variety of force fields [29, 30, 32, 38, 46]. Kinking in the Gly/Pro regions of the portal helices (TM4/6, TM10/12) was observed in the 4M1M system, and the relatively high content of Gly/Pro residues in the TM helices was suggested to lead to enhanced flexibility in P-gp [46]. This observation is in agreement with the kinks in portal helices found in one of the most recent P-gp crystal structures, which extend from Pro219 to Tyr243 [18]. Weaker electron density in the TM4 region, indicating local disorder, has been observed in several crystallographic models of mouse P-gp [17, 18], and in the *C. merolae* P-gp structure [14]. A triple mutation (G277V/A278V/A279V) in the *C. merolae* P-gp improved the resolution from 2.75 Å in the original dataset to 2.6 Å in the mutant, but it was associated with reduced drug transport and ATPase activities. This result suggests that reduction of the flexibility in this region affects protein function.

The majority of the previous simulation studies of P-gp have involved limited sampling and simulation times in the order of 100 ns or less. Most have used the 3G5U model as the starting point. This study found significant divergence in the structural properties between the nine replicas initiated from three different models on a 200 ns time scale. Some structural changes were rapid, such as the NBD association, while the partial unfolding in the TMDs was a continuous process throughout the entire simulation. It is possible that the extent of these deformations would not be evident in shorter simulations. Even at 100 ns, the structural perturbations observed in all three systems could potentially be interpreted as the intrinsic flexibility of the protein. Previous studies have suggested that the extent to which the structural integrity of a protein was maintained during an MD simulation might be used as a potential indicator of the quality of a membrane protein structure [52, 53]. Among the structures examined, the TMD of the 4M1M model showed the highest degree of convergence and stability under the simulated conditions. However, the hinging motion of the NBDs meant the final conformations were further from the initial state. The other two structures (3G5U, 4KSB) showed a higher degree of unfolding in the TMDs, but the TMD-NBD contact was more stable. The intrinsic structural plasticity and divergent behavior of P-gp observed for all three structural models make it impossible to assert which of these three is the most appropriate structural model for future studies. Variations arising from different parameters used in the setup, including the choice of the force field, lipids, salt concentration, pH, temperature, and others, make this task even more complex. Clearly, in the case of P-gp, the results obtained from short simulations of this flexible membrane protein relying on low resolution data should be interpreted with caution.

## Methods

### System setup

The starting configurations of the three P-glycoprotein structures for the MD simulations were taken from the murine P-gp crystal structures corresponding to the PDB IDs: 3G5U [15], 4M1M [16], and 4KSB [17] resolved to 3.8 Å in the absence of any ligands. In the 3G5U and 4M1M structural models, which contain two molecules of P-gp in the asymmetric unit, molecule A was used to initiate the simulations. The protonation states of all the titratable residues were set at their default values at pH 7.0, except for His149, His583, His 1228, which were protonated as reported by [29]. The N- and C-termini of all three structures were acetylated and aminated, respectively. In all three structural models, the linker region of approximately 60 residues connecting the two halves of P-gp was not resolved, and thus, was not included in the simulations. P-gp was embedded in a pre-equilibrated cholesterol enriched POPC (2-oleoyl-1-palmitoyl-sn-glycero-3-phosphocholine) bilayer. The parameters for POPC were taken from [64] and the parameters for cholesterol were obtained from [29]. The membrane used to build the 3G5U and 4M1M systems contained 440 POPC and 40 cholesterol molecules, while the 4KSB P-gp structure was embedded in a larger membrane (660 POPC, 66 cholesterol) to accommodate the larger separation of NBDs. Cholesterol was distributed evenly throughout both leaflets of the bilayer prior to membrane equilibration. Cholesterol distribution around P-gp at the beginning and the end of simulations for each simulated system is shown in S9, S10 and S11 Figs. All simulation systems were solvated using the SPC water model [65]. An electrolyte concentration of 150 mM NaCl and 1.5 mM MgCl_2_ was added to each system to mimic physiological salt concentrations. Additional chloride ions were added to neutralize each system. The total size for 3G5U/4M1M systems was ∼ 235 000 atoms, while 4KSB contained ∼ 340 000 atoms.

### Simulation parameters

All molecular dynamics simulations were performed using GROMACS version 3.3.3 [66] in conjunction with the GROMOS 54A7 force field for proteins [67]. All simulations were performed under periodic boundary conditions in a rectangular box. The dimensions of the box were chosen such that the minimum distance of the protein to the box wall was at least 1.0 nm. A twin-range method was used to evaluate the non-bonded interactions. Interactions within the short-range cut-off of 0.8 nm were updated every step. Interactions within the long-range cut-off of 1.4 nm were updated every 8 fs, together with the pair list. A reaction field correction was applied using a relative dielectric constant of *ϵ*_*r*_ = 78.5, to minimize the effect of truncating the electrostatic interactions beyond the 1.4 nm long-range cut-off [68]. The LINCS algorithm [69] was used to constrain the lengths of all the covalent bonds (*all-bonds*). The geometry of the water molecules was constrained using the SETTLE algorithm [70]. In order to extend the timescale that could be simulated, explicit hydrogen atoms in the protein were replaced with virtual sites, the positions of which were calculated each step based on the positions of the heavy atoms to which they were attached. This eliminates high frequency degrees of freedom associated with the bond angle vibrations involving hydrogens, allowing a time step of 4 fs to be used to integrate the equations of motion without affecting thermodynamic properties of the system significantly [71]. The simulations were carried out in the NPT ensemble at T = 300 K, and P = 1 bar. The temperature was coupled to an external bath using the Berendsen thermostat with a relaxation time constant of 0.1 ps [72]. The Berendsen scheme was also used for semi-isotropic pressure coupling with a relaxation time constant of 0.5 ps. Data was collected every 50 ps during the unbiased MD simulations.

Each system was energy minimized using a steepest descent algorithm. In the 3G5U and 4M1M systems, this was followed by 10 ns of equilibration simulations in which the protein was restrained using a harmonic potential on all heavy protein atoms. These restraints were gradually lowered over 5 consecutive 2 ns simulations, employing force constants of 1000 kJ·mol^−1^·nm^−2^, 500 kJ·mol^−1^·nm^−2^, 200 kJ·mol^−1^·nm^−2^, 100 kJ·mol^−1^·nm^−2^, and 50 kJ·mol^−1^·nm^−2^. In the widely splayed 4KSB system, the position restraints were progressively lowered over a longer period of 30 ns (5 consecutive 6 ns simulations). This longer equilibration time allowed the highly splayed protein conformation of 4KSB P-gp to gradually adjust to the removal of the crystal lattice. After equilibration, new velocities were assigned, and unrestrained MD simulations lasting 200 ns were performed in triplicate for each system.

### Analysis

Trajectory analysis was carried out using the analysis module of the GROMACS package and the MDTraj python toolkit [73]. The root mean squared deviation (RMSD) was calculated using the method of Maiorov and Crippen [74] after first performing a rotational and translational fit of each frame of the trajectory to a reference structure or domain. Pairwise RMSDs were calculated with MDTraj using a concatenated trajectory containing all 9 simulations for the backbone atoms of the entire protein and TMD coordinate subset. Principal component analysis was performed using the ProDy package based on C*α* atoms for the entire system and the TMDs only [75]. Distances and root mean square fluctuations (RMSF) were computed using Gromacs analysis tools. Secondary structure analysis of transmembrane domain was performed using simplified DSSP algorithm implemented in the MDTraj analysis package. In the analysis, the transmembrane domain (TMD) spanned residues Asp46-Phe362 (TMD1) and Val708-Ile1008 (TMD2), while the nucleotide binding domains spanned residues Asn387-Thr626 (NBD1) and Asn1030-Ala1271 (NBD2). Images were produced using VMD [76] and Matlab2015b [77].

### Data availability

All the relevant input files for running MD simulations, as well as resulting trajectories and accompanying videos have been deposited on Figshare (https://doi.org/10.6084/m9.figshare.4806544.v4) [78]. Each trajectory has two accompanying videos showing P-glycoprotein from two different perspectives (*front* and *side*). More detailed description of each movie is provided in an additional document uploaded on Figshare. Model data obtained using principal component analysis are also provided and can be further explored using *loadModel()* function in the Prody package. The extension for these files is *.npz*.

## Supporting information

S1 Fig P-glycoprotein back view. P-glycoprotein with TMD2 (TM7-12) in front depicted using the 4M1M model. The conserved motifs in the NBDs required for ATP binding and hydrolysis, Walker A and Signature motif (LSSGQ) are highlighted in orange and yellow, respectively. The distance between the Walker A1 (located on NBD1) and the Signature motif on NBD2 is labelled as **d1**, and the distance between the Walker A2 (NBD2) and Signature motif 1 (NBD1) is labelled as **d2**. The arrows are pointing towards the Walker A motifs.
(TIFF)

S2 Fig RMSD time series. The RMSD time series measured for the backbone atoms of **(A)** the entire protein, **(B)** transmembrane domain and **(C, D)** the two nucleotide binding domains for each replica, started from the 3G5U (orange), 4KSB (green) and 4M1M (blue) models. All the protein snapshots were aligned to the relevant domain of the reference structure before calculating the RMSD.
(TIFF)

S3 Fig Average RMSD. The mean RMSD values and standard deviations were computed for each independent replica, started from 3G5U (orange), 4KSB (green) and 4M1M (blue). The mean RMSD values are given for the entire protein and each domain separately, namely the two nucleotide binding domains (NBD1, NBD2) and the full transmembrane domain (TMD). The TMD* contains only the TM helices, while the connecting intracellular helices (ICL1-4) and extracellular loops have been removed from these calculations, resulting in lower RMSD values for all three systems.
(TIFF)

S4 Fig Principal component variance. Square fluctuations (variances) corresponding to the first 5 principle components (PC1-5) calculated using a concatenated trajectory containing all 9 simulations started from three different crystal structure models (3G5U, 4KSB, 4M1M). The upper panel shows the principal components calculated for the entire protein, while the lower panel shows the principal components obtained from the analysis of the TMDs only (residues Asp46-Phe362 and Val708-Ile1008). The highest fluctuations in the protein correspond to the NBD movement, while in the TMDs the highest fluctuations are found in the intracellular helices ICL1 (connecting TM2-TM3) and ICL4 (connecting TM10-TM11), which form an interface with NBD1. The extracellular loop connecting TM1 and TM2 is also very dynamic.
(TIFF)

S5 Fig Fraction of variance. Fraction of variance corresponding to the first 10 principal components (PC1-10) calculated using each simulation independently (coloured), and a concatenated trajectory containing all 9 simulations started from the three different crystal structure models (black) for **(A)** the entire protein and **(B)** only the TMDs. The concatenated trajectory yields similar values for the first three PCs for both the total protein and the TMDs only: 34%, 15%, and 10%, respectively. There is greater variability between the PCs resulting from the analysis of each independent simulation. Subspace overlap between the PCs obtained from each replica and the concatenated trajectory is given in S1–S3 Tables.
(TIFF)

S6 Fig RMSF. Root mean squared fluctuations (RMSF) of C*α* atoms in P-gp calculated using data points from three trajectories generated for each system: 3G5U (orange), 4KSB (green) and 4M1M (blue). The upper panel shows the RMSF of C*α* atoms of the entire protein, while the lower panel shows RMSF for the TMD only. The highest fluctuations correspond to NBDs and the intracellular loops forming the interface between TMD and NBDs. The portal helices TM4/6 and TM10/12 have higher RMSF compared to the other helices.
(TIFF)

S7 Fig Secondary structure analysis. Secondary structure analysis of TMD1 (TM1-6) and TMD2 (TM7-12) calculated using a simplified version of the DSSP algorithm implemented in the MDTraj package. In the simplified version, only helical (blue), coil (cyan) and strand (yellow) elements are assigned. White vertical lines separate results obtained from each replica performed for the 3G5U, 4KSB and 4M1M systems, while the length of each TM helix is indicated on the right axis.
(TIFF)

S8 Fig Domain distances. (**A-C**) Distances between the Walker A motif (GxxGxGKS) on one NBD and the signature motif (LSGGQ) located on the opposing NBD (shown as d1 and d2 on S1 Fig). (**D-F**) Distances between NBDs and the intracellular helices at the NBD-TMD interface during triplicate simulations of the 3G5U, 4KSB and 4M1M system.
(TIFF)

S9 Fig Cholesterol distribution in the 3G5U system. Cholesterol distribution around P-glycoprotein in the simulations based on the 3G5U model at (**A**) the beginning of the simulations and at 200 ns for each replica: (**B**) 3G5U #1, (**C**) 3G5U #2, and (**D**) 3G5U #3. The cholesterol molecules are shown in dark violet (upper leaflet) and light violet (lower leaflet) space filling representation. Only the TMD of the protein is shown for clarity.
(TIFF)

S10 Fig Cholesterol distribution in the 4KSB system. Cholesterol distribution around P-glycoprotein in the simulations based on the 4KSB model at (**A**) the beginning of the simulations and at 200 ns for each replica: (**B**) 4KSB #1, (**C**) 4KSB #2, and (**D**) 4KSB #3. The cholesterol molecules are shown in dark violet (upper leaflet) and light violet (lower leaflet) space filling representation. Only the TMD of the protein is shown for clarity. Please note that the simulations of the 4KSB model involved a larger bilayer, the ratio of POPC:cholestorol is the same as in the 3G5U and 4M1M simulations.
(TIFF)

S11 Fig Cholesterol distribution in the 4M1M system. Cholesterol distribution around P-glycoprotein in the simulations based on the 4M1M model at (**A**) the beginning of the simulations and at 200 ns for each replica: (**B**) 4M1M #1, (**C**) 4M1M #2, and (**D**) 4M1M #3. The cholesterol molecules are shown in dark violet (upper leaflet) and light violet (lower leaflet) space filling representation. Only the TMD of the protein is shown for clarity.
(TIFF)

S1 Table Subspace overlap: 3G5U. Pairwise comparison of the 10 principal components obtained for each simulation based on the 3G5U model (3G5U#1-#3 PCs) and the concatenated (3G5U-4KSB-4M1M) trajectory containing all 9 simulations (cPCs). Subspace overlap was calculated as the root mean squared inner product (RMSIP) of every pair of the principal components, as defined in [79] and implemented in the Prody package [75].
(CSV)

S2 Table Subspace overlap: 4KSB. Pairwise comparison of the 10 principal components obtained for each simulation based on the 4KSB model (4KSB#1-#3 PCs) and the concatenated (3G5U-4KSB-4M1M) trajectory containing all 9 simulations (cPCs). Subspace overlap was calculated as the root mean squared inner product (RMSIP) of every pair of the principal components, as defined in [79] and implemented in the Prody package [75].
(CSV)

S3 Table Subspace overlap: 4M1M. Pairwise comparison of the 10 principal components obtained for each simulation based on the 4M1M model (4M1M#1-#3 PCs) and the concatenated (3G5U-4KSB-4M1M) trajectory containing all 9 simulations (cPCs). Subspace overlap was calculated as the root mean squared inner product (RMSIP) of every pair of the principal components, as defined in [79] and implemented in the Prody package [75].
(CSV)

S4 Table Covariance overlap. The covariance overlap between each pair of the 9 replicas and the concatenated trajectory calculated for the entire protein and the TMD coordinate subset only. The covariance overlap was calculated as defined in [80] and implemented in the Prody package [75]. This measure asses the similarity of the spaces sampled by two different trajectories and it ranges from 0, corresponding to no similarity between the fluctuations, to 1, where the fluctuations are identical. The measured covariance overlap between different replicas range between 0.1-0.2.
(CSV)

S1 Video 3G5U #1 trajectory—Front view. The protein movement in the 200 ns trajectory based on the 3G5U model (replica #1). P-glycoprotein is shown in the *front* view depicting the Λ-conformation with TMD1 in the centre, NBD1 on the right side and NBD2 on the left. Frames shown in the video were taken every 100 ps. Corresponding videos for the replicas 3G5U #2 and 3G5U #3 are provided on Figshare: https://doi.org/10.6084/m9.figshare.4806544.v4 [78].
(MP4)

S2 Video 3G5U #1 trajectory—Side view. The protein movement in the 200 ns trajectory based on the 3G5U model (replica #1). P-glycoprotein is shown in the *side* view with the TMD1 and NBD1 in front and with the closest lipids (purple) interacting with TM4/TM6 (left) and TM10/TM12 (right) portals. Frames shown in the video were taken every 100 ps. Corresponding videos for the replicas 3G5U #2 and 3G5U #3 are provided on Figshare: https://doi.org/10.6084/m9.figshare.4806544.v4 [78].
(MP4)

S3 Video 4KSB #1 trajectory—Front view. The protein movement in the 200 ns trajectory based on the 4KSB model (replica #1). P-glycoprotein is shown in the *front* view depicting the Λ-conformation with TMD1 in the centre, NBD1 on the right side and NBD2 on the left. Frames shown in the video were taken every 100 ps. Corresponding videos for the replicas 4KSB#2 and 4KSB #3 are provided on Figshare: https://doi.org/10.6084/m9.figshare.4806544.v4 [78].
(MP4)

S4 Video 4KSB #1 trajectory—Side view. The protein movement in the 200 ns trajectory based on the 4KSB model (replica #1). P-glycoprotein is shown in the *side* view with the TMD1 and NBD1 in front and with the closest lipids (purple) interacting with TM4/TM6 (left) and TM10/TM12 (right) portals. Frames shown in the video were taken every 100 ps. Corresponding videos for the replicas 4KSB #2 and 4KSB #3 are provided on Figshare: https://doi.org/10.6084/m9.figshare.4806544.v4 [78].
(MP4)

S5 Video 4M1M #1 trajectory—Front view. The protein movement in the 200 ns trajectory based on the 4M1M model (replica #1). P-glycoprotein is shown in the *front* view depicting the Λ-conformation with TMD1 in the centre, NBD1 on the right side and NBD2 on the left. Frames shown in the video were taken every 100 ps. Corresponding videos for the replicas 4M1M#2 and 4M1M #3 are provided on Figshare: https://doi.org/10.6084/m9.figshare.4806544.v4 [78].
(MP4)

S6 Video 4M1M #1 trajectory—Side view. The protein movement in the 200 ns trajectory based on the 4M1M model (replica #1). P-glycoprotein is shown in the *side* view with the TMD1 and NBD1 in front and with the closest lipids (purple) interacting with TM4/TM6 (left) and TM10/TM12 (right) portals. Frames shown in the video were taken every 100 ps. Corresponding videos for the replicas 4M1M #2 and 4M1M #3 are provided on Figshare: https://doi.org/10.6084/m9.figshare.4806544.v4 [78].
(MP4)
