# Supplementary figures and images for: The reliability of molecular dynamics simulations of the multidrug transporter P-glycoprotein in a membrane environment
Source: PLoS One. 2018 Jan 25;13(1):e0191882. doi: 10.1371/journal.pone.0191882 (PMC5785007; doi:10.1371/journal.pone.0191882)

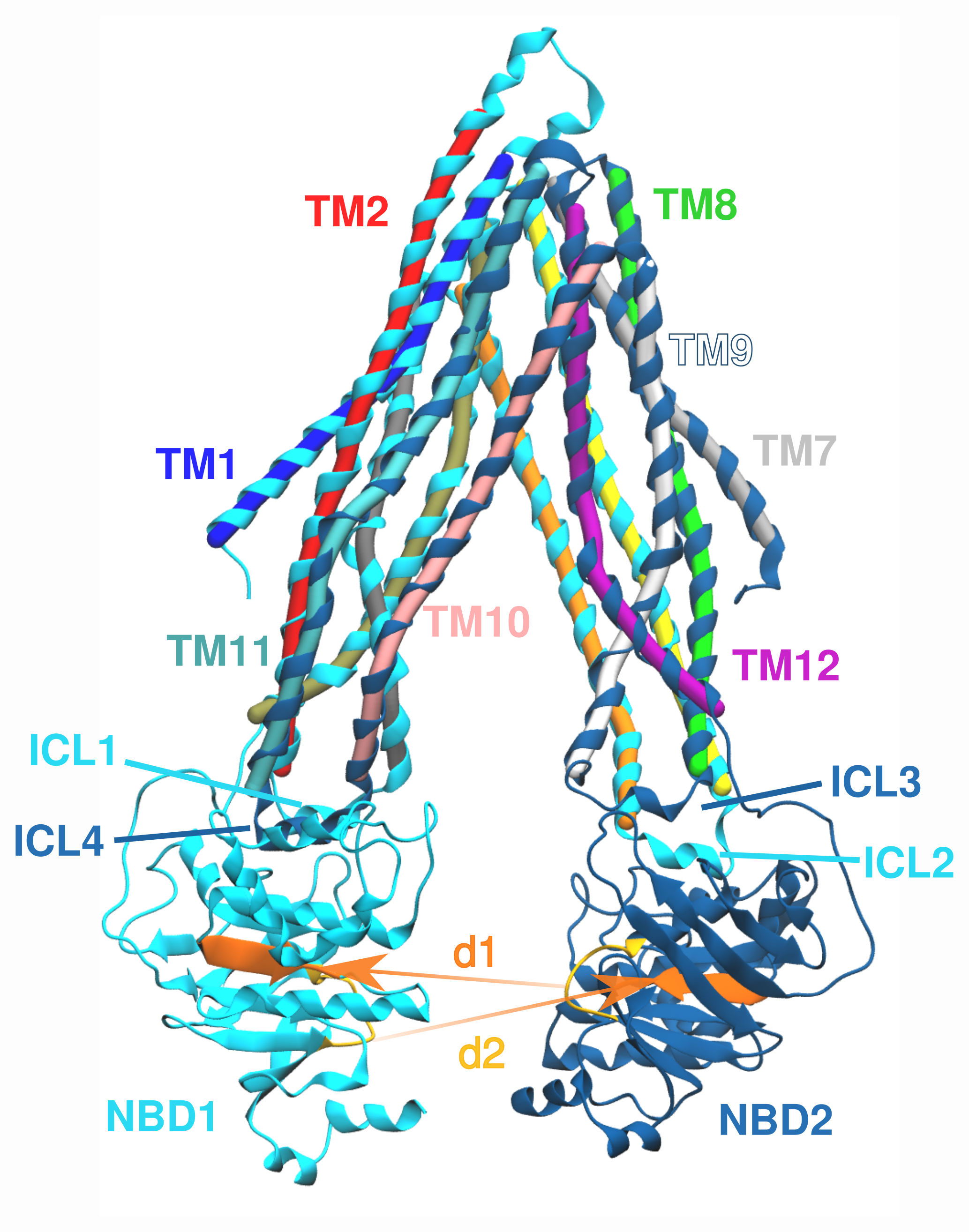

Supplement: S1 Fig — P-glycoprotein with TMD2 (TM7-12) in front depicted using the 4M1M model. The conserved motifs in the NBDs required for ATP binding and hydrolysis, Walker A and Signature motif (LSSGQ) are highlighted in orange and yellow, respectively. The distance between the Walker A1 (located on NBD1) and the Signature motif on NBD2 is labelled as d1, and the distance between the Walker A2 (NBD2) and Signature motif 1 (NBD1) is labelled as d2. The arrows are pointing towards the Walker A motifs. (TIFF) [file pone.0191882.s001.tiff]

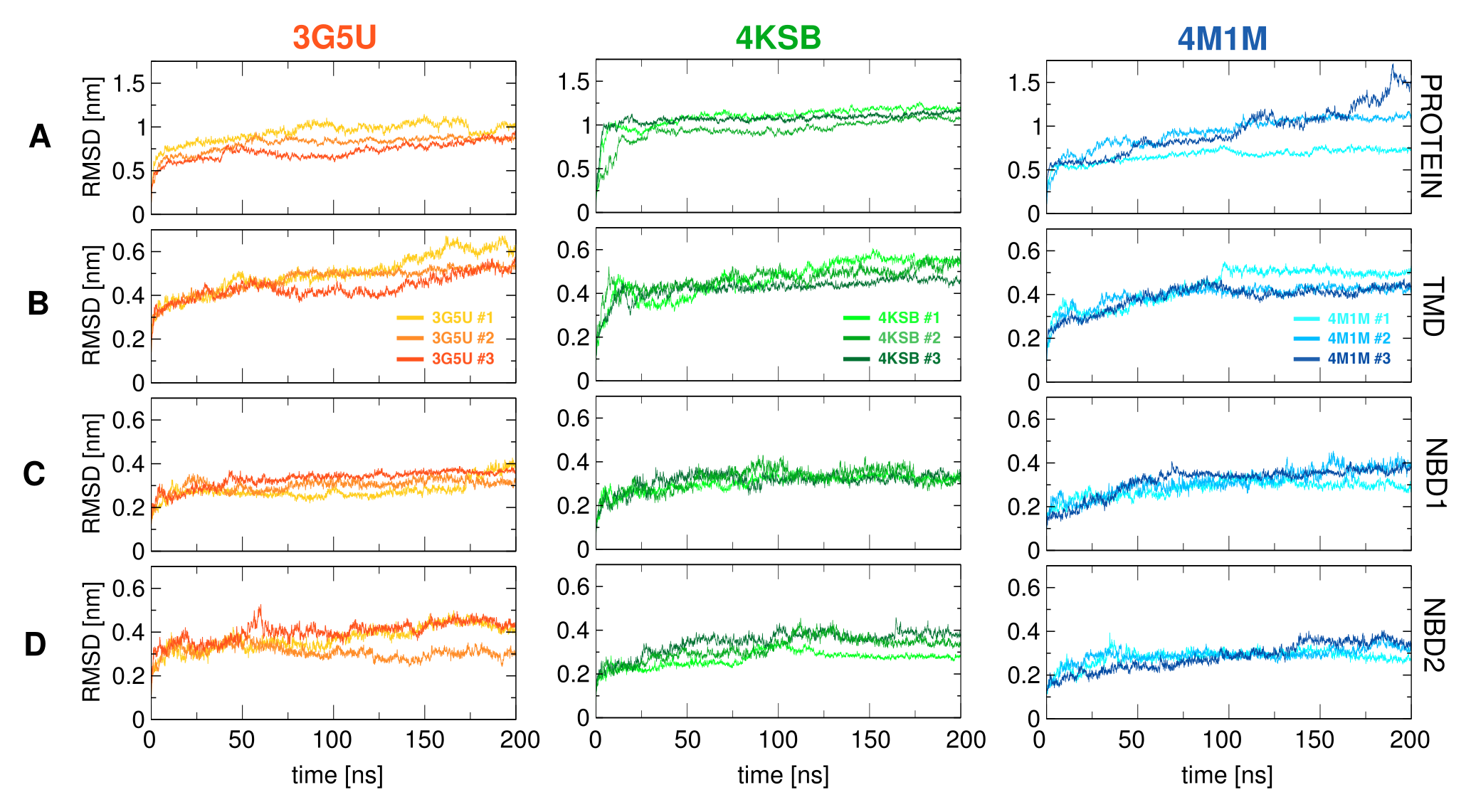

Supplement: S2 Fig — The RMSD time series measured for the backbone atoms of (A) the entire protein, (B) transmembrane domain and (C, D) the two nucleotide binding domains for each replica, started from the 3G5U (orange), 4KSB (green) and 4M1M (blue) models. All the protein snapshots were aligned to the relevant domain of the reference structure before calculating the RMSD. (TIFF) [file pone.0191882.s002.tiff]

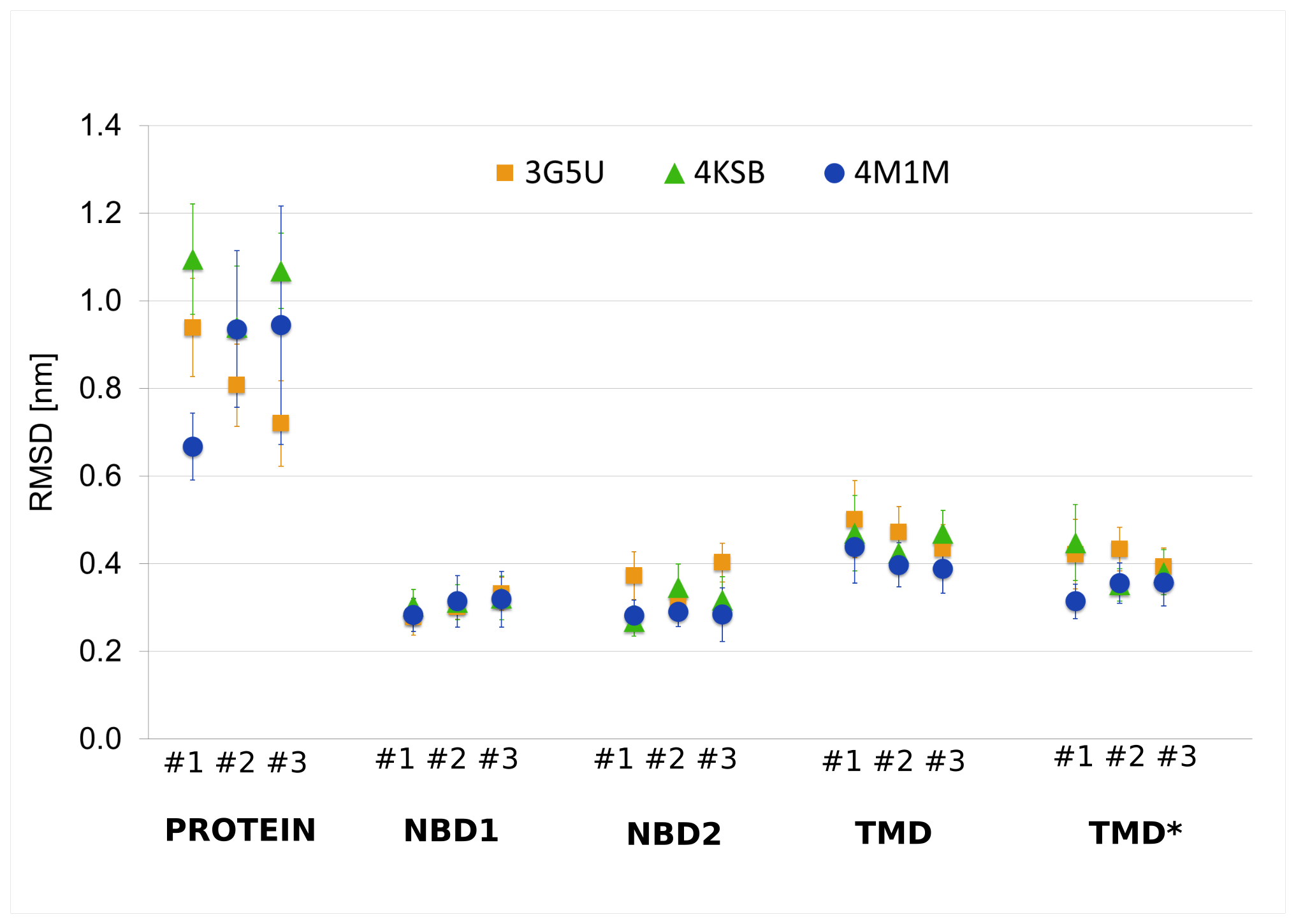

Supplement: S3 Fig — The mean RMSD values and standard deviations were computed for each independent replica, started from 3G5U (orange), 4KSB (green) and 4M1M (blue). The mean RMSD values are given for the entire protein and each domain separately, namely the two nucleotide binding domains (NBD1, NBD2) and the full transmembrane domain (TMD). The TMD* contains only the TM helices, while the connecting intracellular helices (ICL1-4) and extracellular loops have been removed from these calculations, resulting in lower RMSD values for all three systems. (TIFF) [file pone.0191882.s003.tiff]

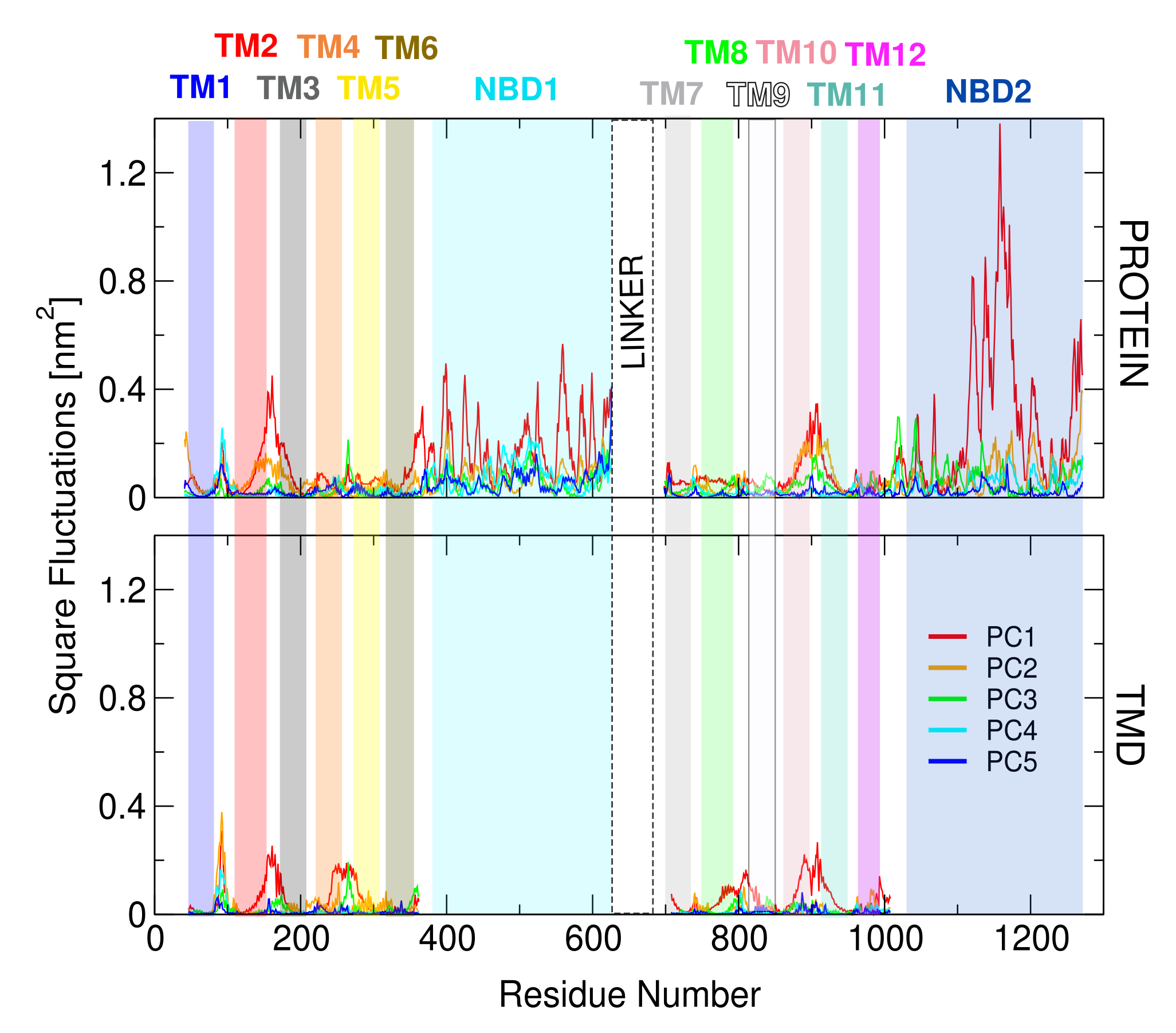

Supplement: S4 Fig — Square fluctuations (variances) corresponding to the first 5 principle components (PC1-5) calculated using a concatenated trajectory containing all 9 simulations started from three different crystal structure models (3G5U, 4KSB, 4M1M). The upper panel shows the principal components calculated for the entire protein, while the lower panel shows the principal components obtained from the analysis of the TMDs only (residues Asp46-Phe362 and Val708-Ile1008). The highest fluctuations in the protein correspond to the NBD movement, while in the TMDs the highest fluctuations are found in the intracellular helices ICL1 (connecting TM2-TM3) and ICL4 (connecting TM10-TM11), which form an interface with NBD1. The extracellular loop connecting TM1 and TM2 is also very dynamic. (TIFF) [file pone.0191882.s004.tiff]

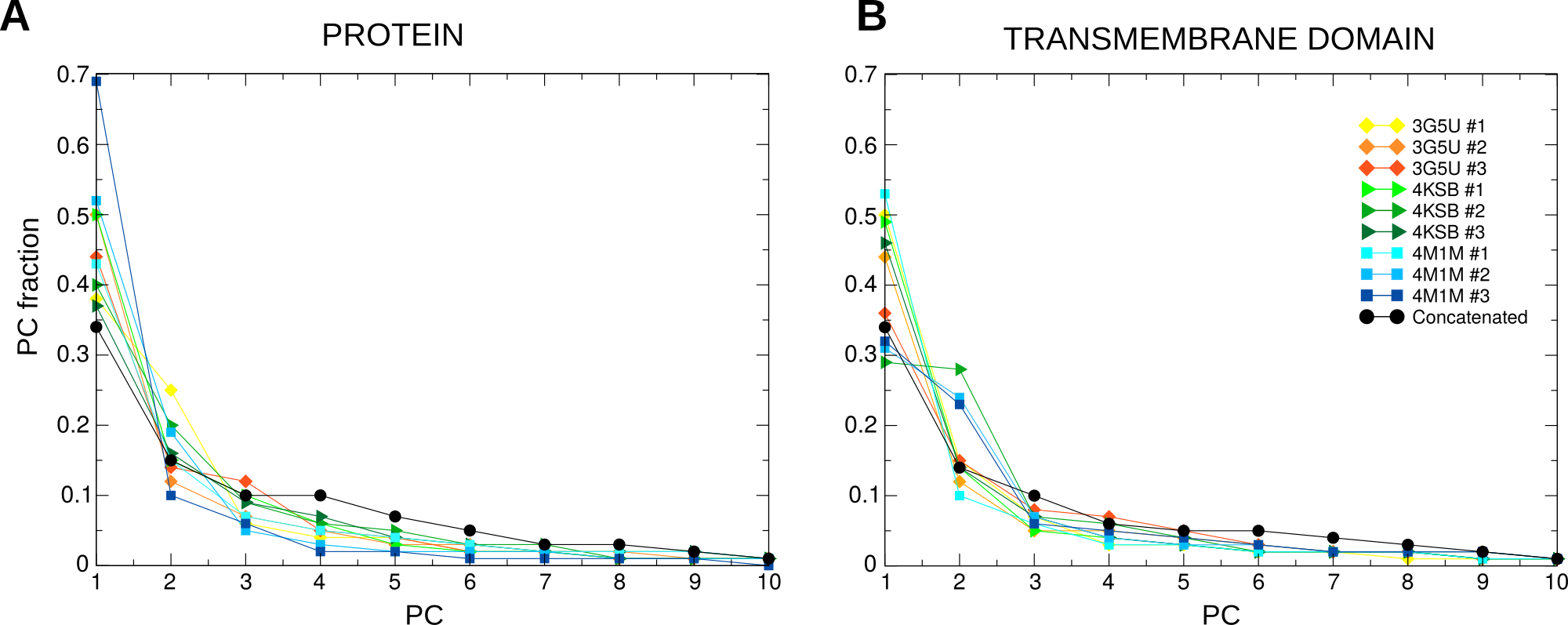

Supplement: S5 Fig — Fraction of variance corresponding to the first 10 principal components (PC1-10) calculated using each simulation independently (coloured), and a concatenated trajectory containing all 9 simulations started from the three different crystal structure models (black) for (A) the entire protein and (B) only the TMDs. The concatenated trajectory yields similar values for the first three PCs for both the total protein and the TMDs only: 34%, 15%, and 10%, respectively. There is greater variability between the PCs resulting from the analysis of each independent simulation. Subspace overlap between the PCs obtained from each replica and the concatenated trajectory is given in S1–S3 Tables. (TIFF) [file pone.0191882.s005.tiff]

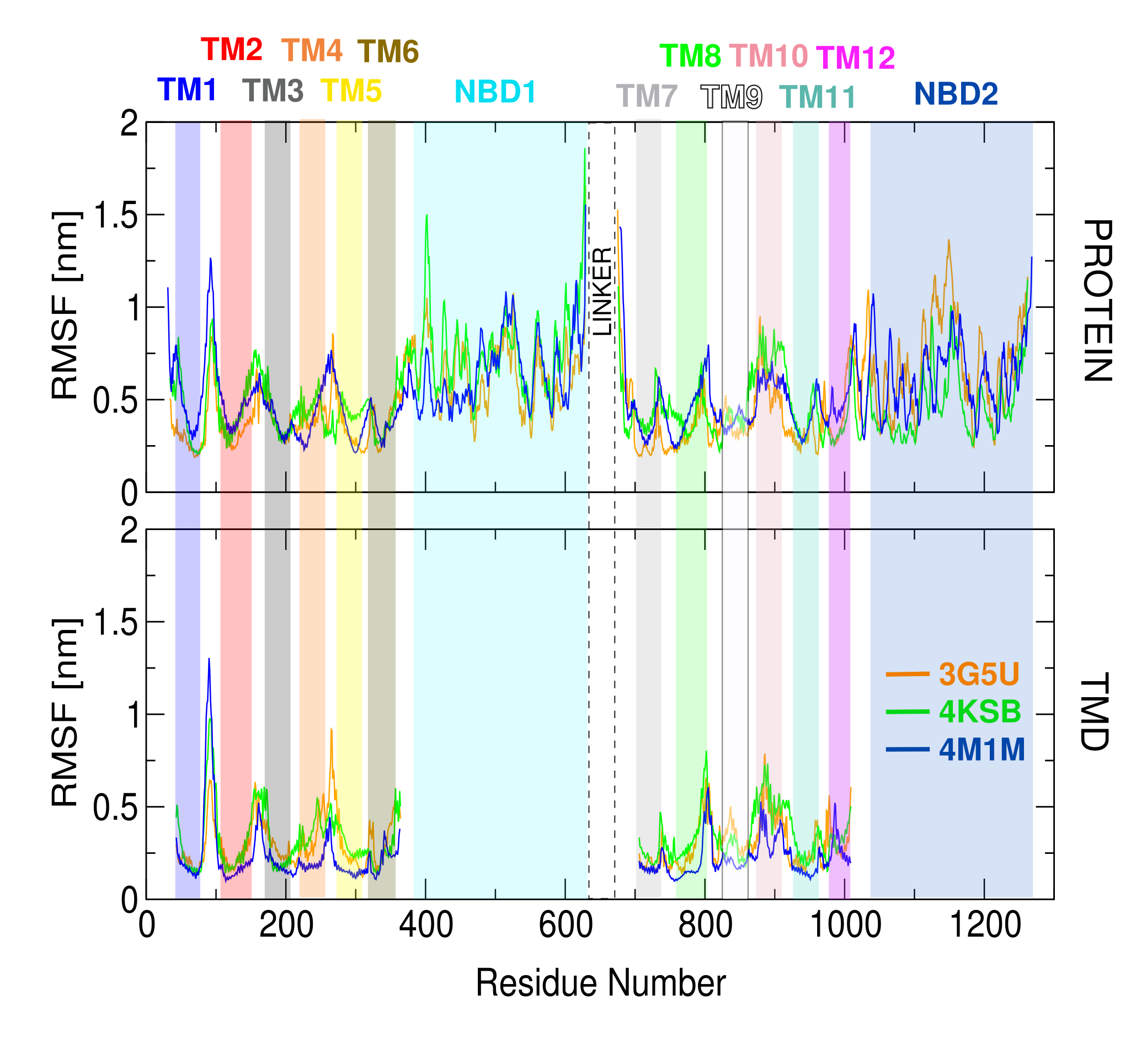

Supplement: S6 Fig — Root mean squared fluctuations (RMSF) of Cα atoms in P-gp calculated using data points from three trajectories generated for each system: 3G5U (orange), 4KSB (green) and 4M1M (blue). The upper panel shows the RMSF of Cα atoms of the entire protein, while the lower panel shows RMSF for the TMD only. The highest fluctuations correspond to NBDs and the intracellular loops forming the interface between TMD and NBDs. The portal helices TM4/6 and TM10/12 have higher RMSF compared to the other helices. (TIFF) [file pone.0191882.s006.tiff]

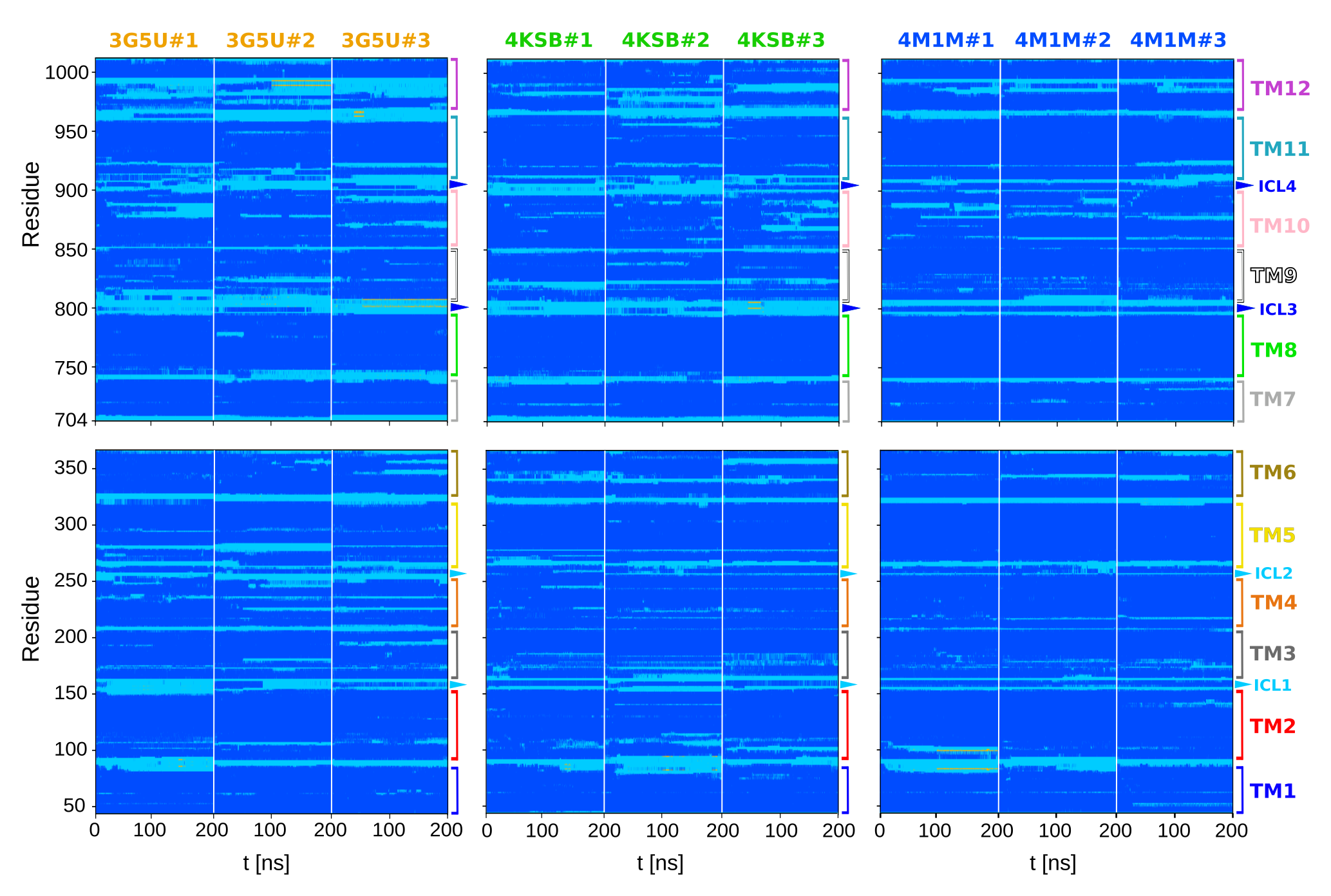

Supplement: S7 Fig — Secondary structure analysis of TMD1 (TM1-6) and TMD2 (TM7-12) calculated using a simplified version of the DSSP algorithm implemented in the MDTraj package. In the simplified version, only helical (blue), coil (cyan) and strand (yellow) elements are assigned. White vertical lines separate results obtained from each replica performed for the 3G5U, 4KSB and 4M1M systems, while the length of each TM helix is indicated on the right axis. (TIFF) [file pone.0191882.s007.tiff]

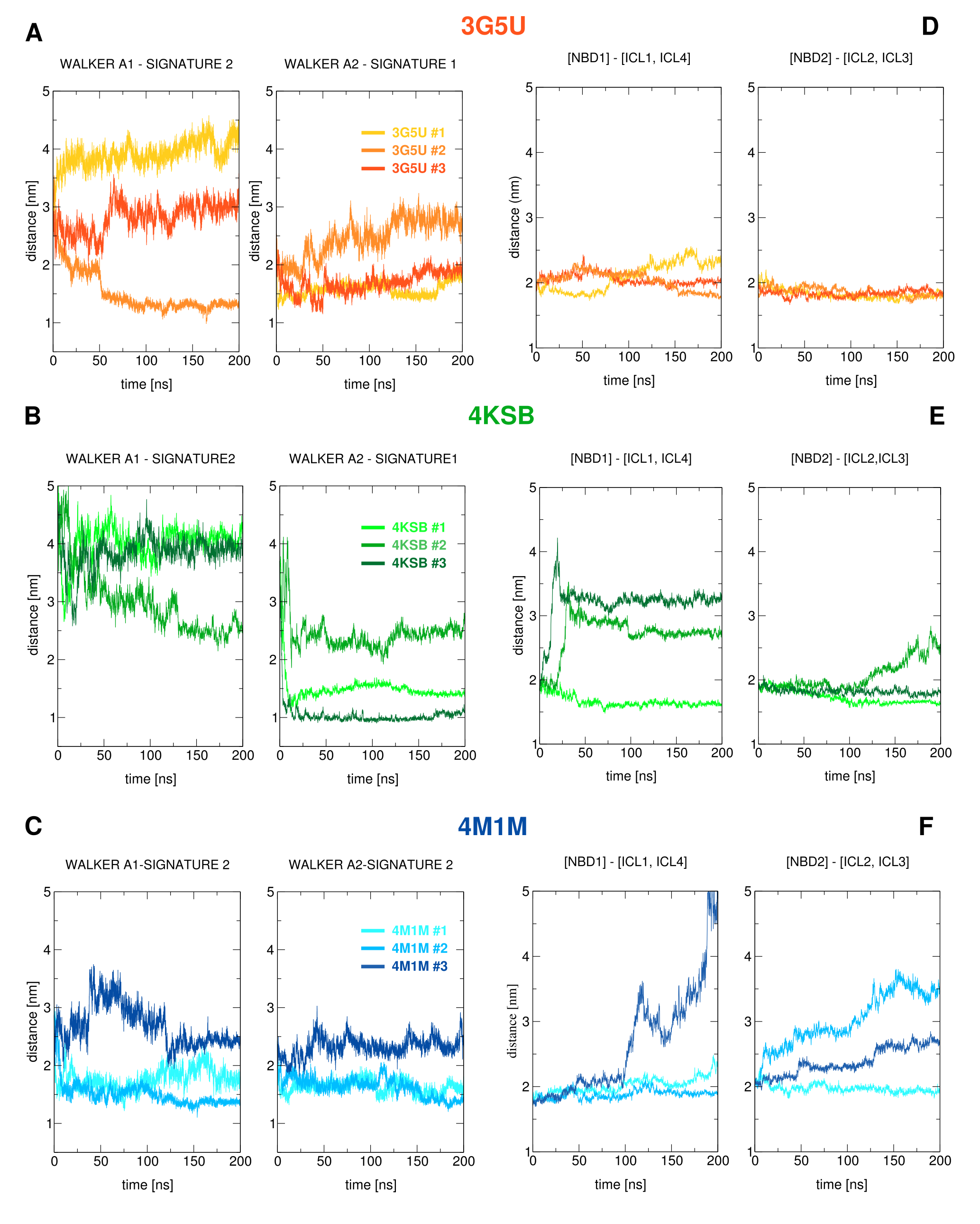

Supplement: S8 Fig — (A-C) Distances between the Walker A motif (GxxGxGKS) on one NBD and the signature motif (LSGGQ) located on the opposing NBD (shown as d1 and d2 on S1 Fig). (D-F) Distances between NBDs and the intracellular helices at the NBD-TMD interface during triplicate simulations of the 3G5U, 4KSB and 4M1M system. (TIFF) [file pone.0191882.s008.tiff]

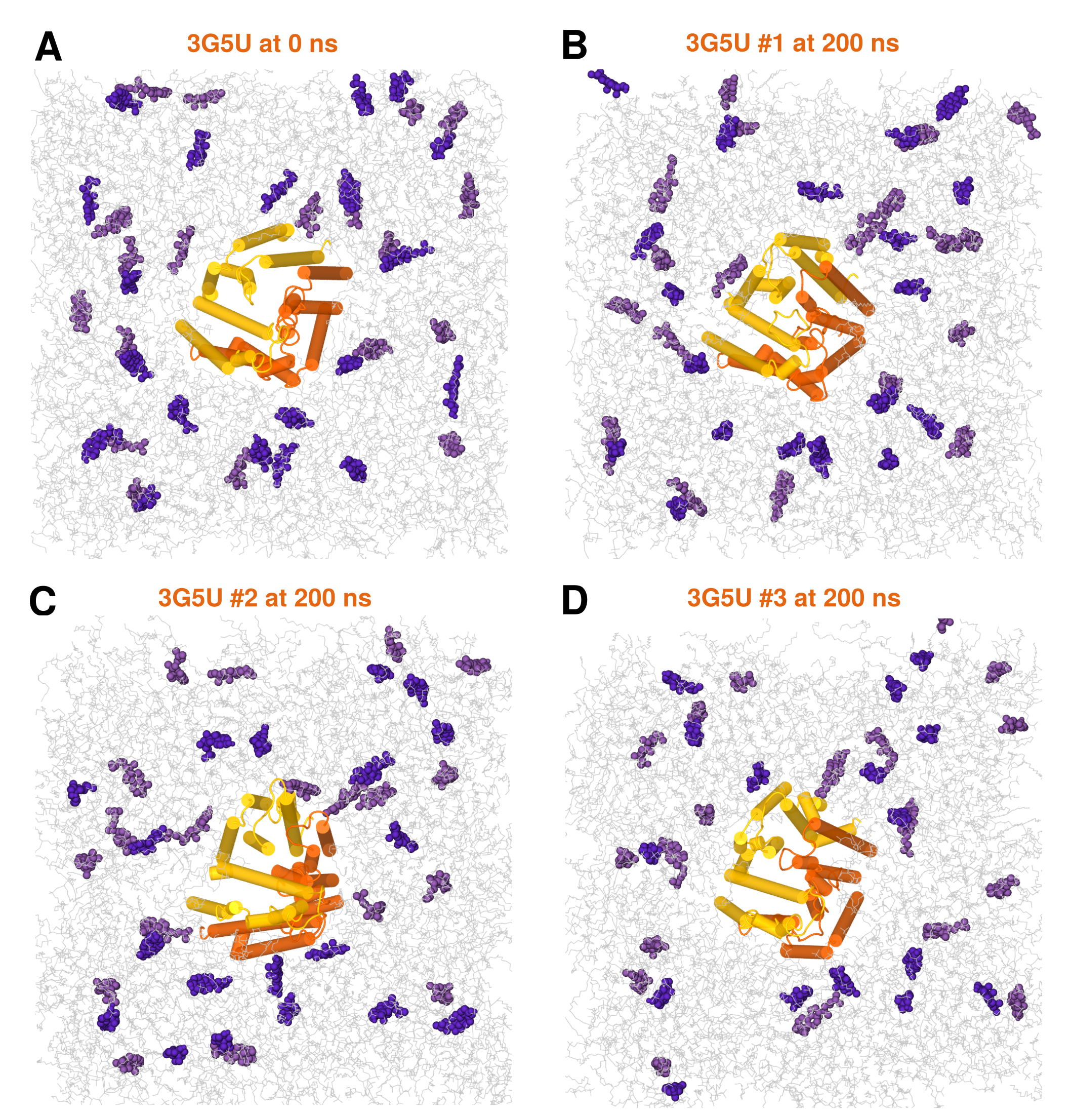

Supplement: S9 Fig — Cholesterol distribution around P-glycoprotein in the simulations based on the 3G5U model at (A) the beginning of the simulations and at 200 ns for each replica: (B) 3G5U #1, (C) 3G5U #2, and (D) 3G5U #3. The cholesterol molecules are shown in dark violet (upper leaflet) and light violet (lower leaflet) space filling representation. Only the TMD of the protein is shown for clarity. (TIFF) [file pone.0191882.s009.tiff]

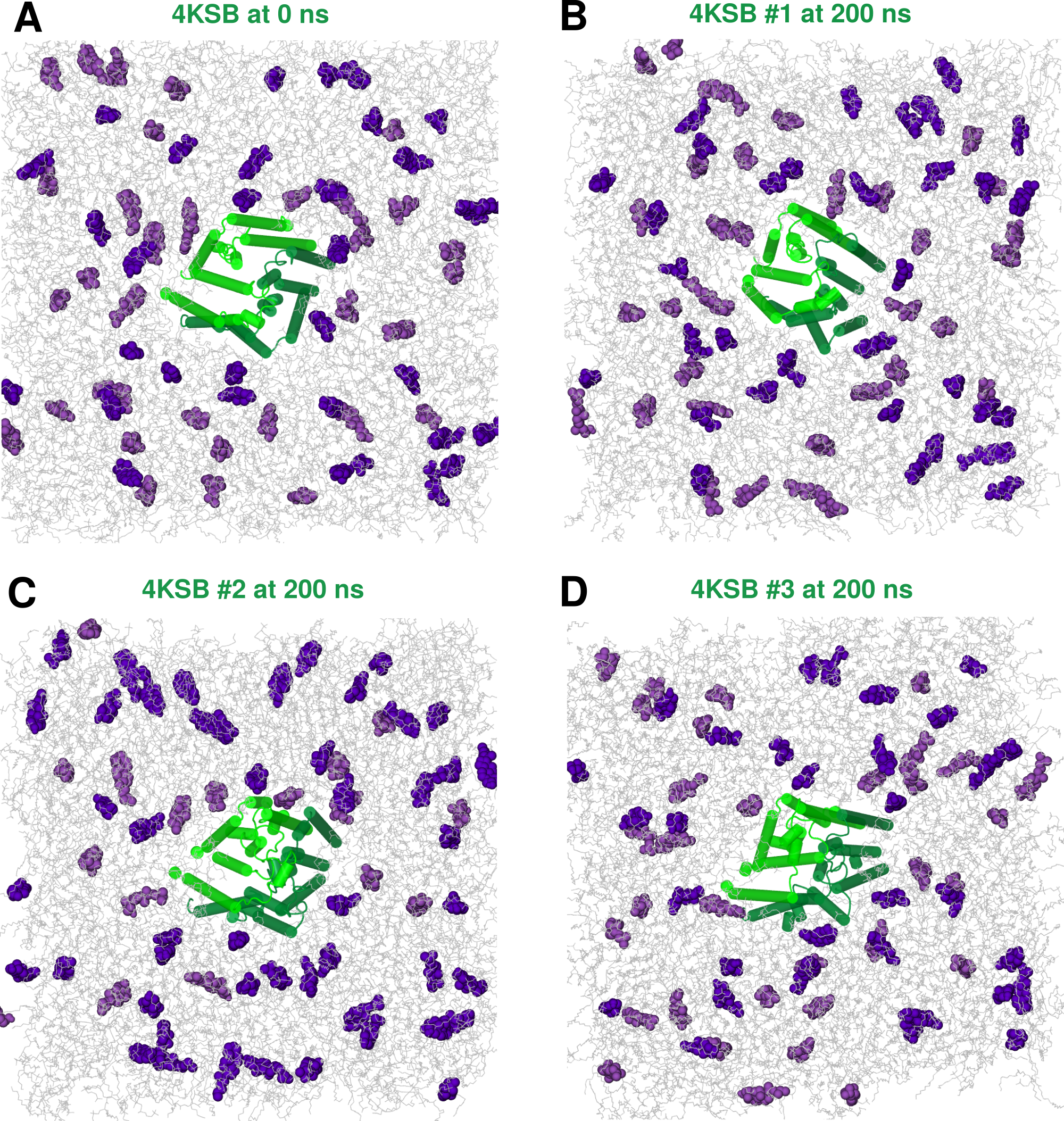

Supplement: S10 Fig — Cholesterol distribution around P-glycoprotein in the simulations based on the 4KSB model at (A) the beginning of the simulations and at 200 ns for each replica: (B) 4KSB #1, (C) 4KSB #2, and (D) 4KSB #3. The cholesterol molecules are shown in dark violet (upper leaflet) and light violet (lower leaflet) space filling representation. Only the TMD of the protein is shown for clarity. Please note that the simulations of the 4KSB model involved a larger bilayer, the ratio of POPC:cholestorol is the same as in the 3G5U and 4M1M simulations. (TIFF) [file pone.0191882.s010.tiff]

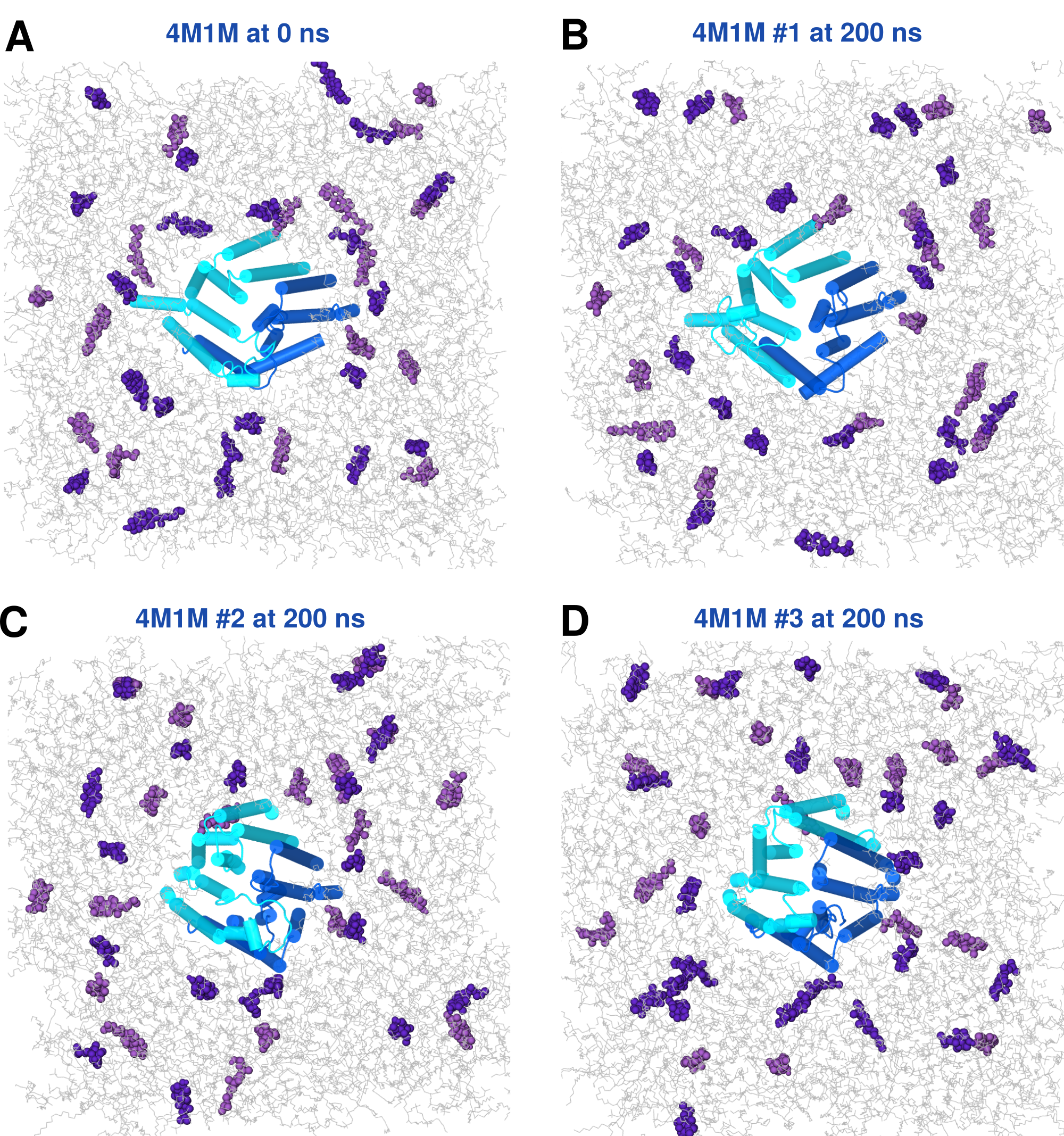

Supplement: S11 Fig — Cholesterol distribution around P-glycoprotein in the simulations based on the 4M1M model at (A) the beginning of the simulations and at 200 ns for each replica: (B) 4M1M #1, (C) 4M1M #2, and (D) 4M1M #3. The cholesterol molecules are shown in dark violet (upper leaflet) and light violet (lower leaflet) space filling representation. Only the TMD of the protein is shown for clarity. (TIFF) [file pone.0191882.s011.tiff]
